# Rapid Characterization of Chemical Components in Edible Mushroom *Sparassis crispa* by UPLC-Orbitrap MS Analysis and Potential Inhibitory Effects on Allergic Rhinitis

**DOI:** 10.3390/molecules24163014

**Published:** 2019-08-20

**Authors:** Zhixin Wang, Jingyu Liu, Xiangjian Zhong, Jinjie Li, Xin Wang, Linlin Ji, Xiaoya Shang

**Affiliations:** 1 Beijing Key Laboratory of Bioactive Substance and Functional Food, Beijing Union University, No.191 Beitucheng West Road, Haidian District, Beijing 100191, China; 2 College of Food Science and Engineering, Shanxi Agricultural University, No. 1 Mingxian South Road, Taigu County, Jinzhong 030801, China

**Keywords:** *Sparassis crispa*, identification, UPLC-MS, allergic rhinitis (AR), anti-inflammatory activity

## Abstract

*Sparassis crispa* is a kind of edible fungus widely grows in the north temperate zone, which shows various medicinal properties. Due to the complexity of chemical constitutes of this species, few investigations have acquired a comprehensive configuration for the chemical profile of it. In this study, a strategy based on ultra-high performance liquid chromatography (UPLC) combined with Orbitrap mass spectrometer (MS) was established for rapidly characterizing various chemical components in *S. crispa*. Through the summarized MS/MS fragmentation patterns of reference compounds and systematic identification strategy, a total of 110 components attributed to six categories were identified for the first time. Moreover, allergic rhinitis (AR) is a worldwide inflammatory disease seriously affecting human health, and the development of drugs to treat AR has been a topic of interest. It has been reported that the extracts of *S. crispa* showed obvious inhibitory effects on degranulation of mast cell- and allergen-induced IgE and proinflammatory mediators, but the active components and specific mechanism were still not clear. Src family kinases (SFKs) participate in the initial stage of allergy occurrence, which are considered the targets of AR treatment. Herein, on the basis of that self-built chemical database, virtual screening was applied to predict the potential SFKs inhibitors in *S. crispa*, using known crystal structures of Hck, Lyn, Fyn, and Syk as receptors, followed by the anti-inflammatory activity evaluation for screened hits by intracellular calcium mobilization assay. As results, sparoside A was directly confirmed to have strong anti-inflammatory activity with an IC_50_ value of 5.06 ± 0.60 μM. This study provides a useful elucidation for the chemical composition of *S. crispa*, and demonstrated its potential inhibitory effects on AR, which could promote the research and development of effective agents from natural resources.

## 1. Introduction

*Sparassis crispa*, namely “cauliflower mushroom”, is a brown-rot fungus that widely grows on the stumps of coniferous trees in the north temperate zone, as northeast of China and Japan. As a kind of valuable edible mushroom, *S. crispa* shows various medicinal properties, such as anti-tumor [1], anti-inflammation [2], immunoregulation [3], hypoglycemic effect [4], and ameliorating skin conditions [5]. A number of previous studies have reported the isolation and structural determination of more than thirty compounds within different categories in this species, including alkaloids [6], phthalides [6,7], sesquiterpenes [8], steroids [9], and other compounds [10]. The genes responsible for the synthesis of these secondary metabolites were known to form clusters. A total of thirty gene clusters were potentially identified in the *S. crispa* genome, among which fifteen genes encoded terpene synthase for the synthesis of sesquiterpenes, two encoded indole prenyltransferase or dimethylallyl tryptophan synthase for the synthesis of indole alkaloids, five encoded type I polyketide synthases for the synthesis of aromatic and highly reduced polyketide metabolites, and eight encoded for other compounds, respectively [11]. However, due to the intrinsic complexity of chemical constitutes of *S. crispa*, few investigations have acquired a comprehensive configuration for the chemical profile of it. Bang et al. [12] developed a HPLC-DAD method to simultaneously quantify the major compounds in extracts of *S. crispa*, but the upstream separation process was still unavoidable and laborious. Gas chromatography mass spectrometer (GC-MS) coupled with multivariate statistical analysis was performed by Seo et al. [13] to understand the difference of metabolites between pileus and stipe of *S. crispa*, as well as the metabolic changes after fermentation by different microorganisms, which provided novel insights into chemical characteristics of different parts of this mushroom. But because of the lack of subsequent peaks identification process, it could not point out the specific components which cause those differences, and chemical components with greater polarity could not be detected. Thus, it is very necessary and challenging to establish an efficient strategy to comprehensively characterize the chemical components of *S. crispa*. In recent decades, based on the highly efficient separation performance of ultra-high performance liquid chromatography (UPLC) and high sensitivity of MS, UPLC tandem MS has already become an important technology for characterization of chemical components in natural product [14]. Especially, UPLC coupled with Orbitrap MS system is applied by more and more labs in this field, because it is ideal for the identification of compounds by obtaining accurate molecular mass and multistage MS^n^ fragment ions of samples to be analyzed [15]. In this study, a strategy based on UPLC combined with Orbitrap MS/MS was established for rapid characterizing various chemical components in *S. crispa*. Through the summarized MS/MS fragmentation patterns of reference compounds and systematic identification strategy, a total of 110 components belonging to six categories in *S. crispa* were rapidly isolated within a short time and identified for the first time, by which the preliminary chemical substance database of *S. crispa* was self-built.

Allergic rhinitis (AR) is a worldwide inflammatory disease seriously affecting human health, characterized by elevated production of IgE and mast cell degranulation that result in the release of histamine and other chemical mediators of allergy [16]. Drug development to treat AR has been a topic receiving great attention, while natural products could provide abundant substance source for this purpose. Interestingly, it has been reported that both serum IgE level and the number of NC/Nga mice with induced dermatitis by a continuous application of hapten scratching were reduced by oral administration of the extract of *S. crispa* [17]. Takashi [17] observed an obvious inhibition effect on allergen-induced IgE and cytokines production by murine splenocytes in ovalbumin-sensitized BALB/c mice fed with *S. crispa*. Han et al. [18] found that the fraction of *S. crispa* isolated by chloroform could significantly inhibit the production of LPS-induced proinflammatory mediators, such as NO and PGE2 and cytokines including TNF-α, and interleukin IL-6 and IL-1β, without cytotoxicity. Yoshikawa et al. [7] found that phthalides isolated from *S. crispa* showed inhibition for PGE2 and NO in LPS-induced mouse macrophage RAW264.7, which was dose-dependent. However, the active components and specific mechanism of *S. crispa* treating AR were still not completely clear. As a group of non-receptor tyrosine kinases, Src family kinases (SFKs) are composed of 11 members including Src, Hck, Syk, Lyn, Fyn, Lck, Fgr, c-yes, Blk, Brk, and Srm, which share similar configuration and the same functional domain and participate in cell transformation and intracellular signal transmission. Many literatures reported that mast cell degranulation is the key process in AR occurrence [19], part of SFKs take part in the initial signal of degranulation, and has both positive and negative regulatory effects [20]. It could be concluded that the aim of treating AR can be achieved by inhibiting SFKs. Currently, some small molecule SFKs inhibitors have been developed for the treatment of AR and other diseases. For example, the Syk inhibitor R112 developed by Rigel company has a good curative effect on seasonal AR and various other allergic disease, and has already entered the clinical trial stage [21]. In the field of natural products, a large number of studies have also found that some natural compounds can regulate mast cell degranulation by inhibiting SFKs, so as to play an anti-allergic role. Lu et al. [22] reported that nujiangexanthone A isolated from garcinia could inhibit IgE/Ag-induced mast cell activation by inhibiting Syk enzyme activity, thereby inhibiting degranulation and the production of eicosanoids. In addition, some other natural compounds including atractylode lactone Ⅲ [23], piceatannol [23], rosmarinic acid [24], emodin [25], and resveratrol [26] all could inhibit mast cell degranulation by inhibiting SFKs. Herein, on the basis of that self-built chemical substance database of *S. crispa*, virtual screening based molecular docking was firstly applied to predict the potential SFKs inhibitors in *S. crispa*, using known crystal structures as targeted receptors including Hck, Lyn, Fyn, and Syk, followed by the anti-inflammatory activity evaluation for screened hits by intracellular calcium mobilization assay. As results, some compounds were fished out by virtual screening as the potential SFKs inhibitors. Among them, sparoside A was directly confirmed to show strong anti-inflammatory activity for the first time, with an IC_50_ value of 5.06 ± 0.60 μM. In conclusion, this study provides a useful elucidation for the chemical composition of *S. crispa*, and demonstrated its potential inhibitory effects on AR, which could promote the development of effective agents from natural resources.

## 2. Results and Discussion

### 2.1. Optimization of UPLC and MS Conditions

In order to acquire chromatograms with intense peak response and high resolution, the mobile phase compositions were firstly optimized. Compared with methanol/water, the acetonitrile/water system showed higher baseline stability and lower pressure, as well as stronger elution and isolation abilities for investigated components. When a small amount of formic acid was added into the water phase, the shapes of most peaks were improved apparently. Therefore, it was finally decided that acetonitrile/0.1% formic acid aqueous solution was used as the mobile phase. The column temperature was set at 40 °C to reduce the pressure, and flow rate was constant at 0.4 mL/min.

To acquire high sensitivity for most analytes, some parameters of heated electrospray ionization (HESI) source were also optimized by multiple experiments, including sheath gas flow, auxiliary gas flow, spray voltage, source heater temperature, capillary temperature, capillary voltage, and tube lens voltage. These parameters directly contributed little to total ion current chromatogram (TIC) but were key for MS/MS fragmentation. The optimal conditions were set as follows: sheath gas flow, 50 arb; auxiliary gas flow, 10 arb; spray voltage, 4 kV/3.5 kV (positive/negative); probe heater temperature, 350 °C; capillary temperature, 380 °C; S-lens RF level, 55. Because the molecular mass of all known compounds in *S. crispa* was distributed in the range of 100–1,500 Da, in full scan mode the mass spectra were acquired in the *m/z* range of 50–1,500 Da, and the resolution was empirically set as 70,000. Moreover, the sizes of collision-induced dissociation (CID) energy were also considered. After some attempts, the MS/MS energy was finally set as 30 V as stepped normalized collision energy (NCE), under which more abundant fragment ions with appropriate mass could be produced at the resolution of 35,000.

### 2.2. UPLC-Orbitrap MS Analysis of S. crispa and Component Identification

**Figure 1:**
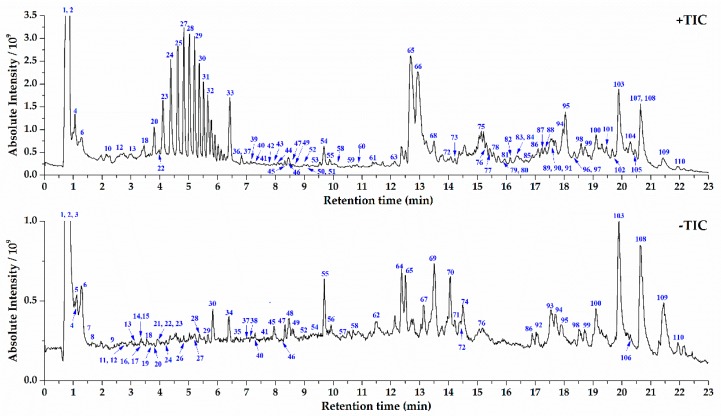
Total ion current chromatogram (TIC) of *Sparassis crispa* extracts in positive and negative ESI modes.

The optimized UPLC and Orbitrap MS conditions were applied for characterization of chemical components in *S. crispa* extracts. The TIC in positive and negative ESI modes were shown in Figure 1. The reported compounds in *S. crispa* could be classified into seven types on the grounds of their chemical structures: alkaloids, organic acid, sterols, sesquiterpenes, sterols, phthalides, and others. Except organic acids, most compounds showed strong response and typical fragmentation in the positive ESI mode. Thus, the targeted MS/MS experiments for citric acid (**2**) were conducted in negative mode, and others were in positive mode. The identification of components in *S. crispa* started from the recognition of part known compounds by importing the data into the Compound Discoverer 2.1 loaded with OTCML database. Then, the unidentified most peaks were processed through an established systematic strategy based on high-resolution MS [14]. First of all, the chemical elemental composition for each targeted peak was deduced by the accurate mass spectra of designated protonated/deprotonated molecular ions or adduct ions using a formula predictor, as well as their corresponding isobaric molecular ions. The proposed molecular formulas were also approved by additional judgements such as nitrogen rule, elemental composition of fragment ions and general formula features of natural compounds. Then the formulas were searched in self-built chemical database of *S. crispa* to match the known structures. For those formulas not included in the self-built database, they could be input into the SciFinder database for screening possible compounds, and the hits were refined in the genus of *Sparassis*. The next process was to verify components after learning the knowledge of characteristic product ions and fragmentation rules of various types of compounds, and the MS/MS fragmentation patterns of six reference compounds were sufficiently investigated (Table 1). Those components owned the identical retention time, mass and fragment ions with the reference compounds were firstly identified undoubtedly. Other components could be identified via comparing the fragmentation patterns with those known analogous compounds and referring reported structures in literatures. Finally, a total of 110 compounds in *S. crispa* extracts were identified or tentatively identified. The retention time, *m/z* values of adduct ions and MS/MS fragment ions in positive/negative ESI modes, mass error, accurate molecular mass, formula, and confidence levels of identity [27] of all the identified compounds were completely summarized in Table S1.

### 2.3. Structural Characterization and Identification of Various Type of Components in S. crispa

#### 2.3.1. Structural Characterization and Identification of Alkaloids

One of the representative alkaloids, riboflavin (**1**, peak 22) [28], was selected as a reference compound to investigate the MS/MS fragmentation patterns of alkaloids in *S. crispa* (see Figure S1). The protonated molecular ion *m*/*z* 377.1458 [M + H]^+^ of riboflavin could be easily formed in positive ion mode, and it dehydrated (losing H_2_O) to form [M – 18 + H]^+^ fragment ion of *m*/*z* 359.1349; and then its secondary dehydration produced [M – 36 + H]^+^ fragment ion of *m*/*z* 341.1245. Fragment ion *m*/*z* 243.0876 [M – 134 + H]^+^ with the highest relative abundance was easily produced from *m*/*z* 341.1245 by cleavage of entire side C-chain, which could be also produced from *m*/*z* 377.1458 and *m*/*z* 359.1349 directly. Fragment ion *m*/*z* 99.0445 [M – 144 + H]^+^ was derived from the cyclization of the detached side C-chain. In a similar way, the other 33 alkaloids were identified according to their molecular mass, formula, MS/MS fragments, and related literatures, including arginine (peak 1) [29], ethyl-l-glutaminyl-l-threonine (peak 4) [30], 5′-deoxy-5′-methylthioadenosine (peak 12) [6], leucylproline (peak 13) [31], 2-aminooctanedioic acid (peak 16) [32], 2-amino-1,3-dodecanediol (peak 36) [33], stellarin C (peak 37) [34], 2-amino-1,3,4-tetradecanetriol (peak 39) [35], tuberostemonine (peak 40) [36], salternamide d (peak 41) [37], 3-(acetyloxy)-17-(benzoyloxy)-7-[*O*-(phenylmethyl)oxime] androst-5-en-7-one (peak 45) [38], antrodin d (peak 50) [39], 1-(18-benzamido-3β-hydroxy-5α-androstan-17β-yl)-3-phenyl-2-propen-1-one (peak 52) [40], 3-cyclohexene-1-butyraldehyde-3-hydroxy-α-isopropyl-1-methyl-2-oxo-disemicarbazone (peak 54) [41], antrodin C (peak 59) [39], 2-amino-1,3,4-trihydroxyoctadecane (peak 66) [42], 2-amino-1,3-hexadecanediol (peak 77) [43], stachybotrin G (peak 78) [44], 2-methyl-6-(11-oxododecyl)piperidin-3-yl acetate (peak 82) [30], 2-methyl-6-(13-oxotetradecyl)piperidin-3-yl acetate (peak 86) [30], oleoylethanolamide (peak 87) [45], hurghamide D (peak 88) [46], 2-amino-1,3,4-octadecanetriol (peak 91) [47], oleamide (peak 94) [48], hexadecanamide (peak 97) [49], veracintine (peak 99), 2-nonadecananone-*O*-methyloxime (peak 101) [50], 4-azacholest-5-en-3-one (peak 102) [51], N-(2-phenylethyl)hexadecanamide (peak 103) [52], stearamide (peak 104) [53], thraustochytroside A (peak 105) [54], rhizoleucinoside (peak 106) [55], and erucamide (peak 107) [56]. Among these alkaloids, except 5′-deoxy-5′-methylthioadenosine, antrodin D, and antrodin C, all other 31 compounds were identified from the genus of *Sparassis* for the first time.

#### 2.3.2. Structural Characterization and Identification of Organic Acids

As shown in Figure S2, the deprotonated molecular ion of citric acid (**2**, peak 3) [56] was observed at *m*/*z* 191.0195 [M − H]^-^ in negative ion mode, which could easily yield the MS/MS fragment ion *m*/*z* 173.0089 [M – 18 − H]^-^ after dehydration, and then the latter one could yield the fragment *m*/*z* 129.0192 [M – 62 − H]^-^ through decarboxylation (losing CO_2_). After that, by dehydration, the smaller but most abundant fragment ion *m*/*z* 111.0086 [M – 80 − H]^-^ was produced from the fragment *m*/*z* 129.0192, or by the secondary decarboxylation at a higher collision energy, the fragment *m*/*z* 82.0295 [M – 62 − H]^-^ was formed. In this way, other 22 organic acids were identified, including mevalonic acid (peak 7) [57], methylsuccinic acid (peak 8) [58], 3-methylglutaric acid (peak 9) [59], suberic acid (peak 26) [60], 3-tertbutyladipic acid (peak 38) [61], 9-hydroxy-10,14-octadecadien-12-ynoic acid (peak 43) [62], 9,12,13-trihydroxy-15-octadecenoic acid (peak 48) [63], moroctic acid (peak 49) [64], 5,8-dihydroxy-9,12-octadecadienoic acid (peak 55) [63], 3-hydroxy-(4,2-hydroxy-6-methyl)-2 heptanyl benzoic acid (peak 57) [65], porrigenic acid (peak 58) [66], 12-oxo-phytodienoic acid (peak 60) [67], 3-methyl-5-pentyl-2-furanundecanoic acid (peak 63) [68], 10-hydroxy-8,12-octadecadienoic acid (peak 67), α-eleostearic acid (peak 68) [69], 8-oxo-9-octadecenoic acid (peak 69) [70], 6,7-epoxystearic acid (peak 71) [63], 9-oxo-octadecadienoic acid (peak 73) [71], 2,3-seco-2,3-dicarboxyplatanic acid (peak 74) [72], diroleuton (peak 83) [73], linoleic acid (peak 93) [74], and 1-(hydroxymethyl)-2[(1-oxohexadecyl)oxy]ethyl-ester-9,12-heptadecadienoic acid (peak 108) [75]. All these organic acids were identified from the genus of *Sparassis* for the first time.

#### 2.3.3. Structural Characterization and Identification of Sesquiterpenes

Six sesquiterpenes were identified from the extracts of *S. crispa*, including clitocybulol C (peak 46) [76], 3β-hydroxy-11,12-*O*-isopropylidenedrimene (peak 47) [77], ustusol B (peak 56) [78], 3-(10-hydroxy-10-methylethyl)-5,8a-dimethyldecahydroazulen-4-ol (peak 109) [8], and (5β, 6α)-6,11-dihydroxyeudesmane (peak 110) [8]. As shown in Figure S3, in positive ESI mode, the protonated molecular ion of ainsliatone (**3**, peak 25) [79] A was *m*/*z* 251.1277 [M + H]^+^, and its intramolecular dehydration produced the fragment ion *m*/*z* 233.1174 [M – 18 + H]^+^. The following continuous dehydration of fragment ion *m*/*z* 233.1174 could form the fragments *m*/*z* 215 [M – 36 + H]^+^ and *m*/*z* 197 [M – 54 + H]^+^. Due to the instability of the lactonic ring, it was easily opened and lost one formic acid (HCOOH), resulting in the fragment ion *m*/*z* 205.1231 [M – 46 + H]^+^, which was followed by the two steps of dehydration and produced the fragments *m*/*z* 187.1118 [M – 64 + H]^+^ and *m*/*z* 169.1013 [M – 82 + H]^+^. Moreover, the oxygen-free fragment ion 159.1170 [M – 92 + H]^+^ and 145.1012 [M – 106 + H]^+^ was formed by decarbonylation (losing CO) and ring opening-deoxygenation reaction from fragments *m*/*z* 187.1118, respectively. Among those above identified sesquiterpenes, ainsliatone A, clitocybulol C, 3β-hydroxy-11,12-*O*-isopropylidenedrimene, and ustusol B were identified from the genus of *Sparassis* for the first time.

#### 2.3.4. Structural Characterization and Identification of Sterols

A typical sterol, ergosterol (**4**, peak 98) [8], was taken as an example to investigate the MS/MS fragmentation pattern of this type of compounds in *S. crispa* (see Figure S4). The protonated molecular ion of ergosterol was *m*/*z* 397.3460 [M + H]^+^ in positive ESI mode, and its dehydration of C_1_-OH with adjacent hydrogen could easily yield the fragment ion *m*/*z* 379.3359 [M – 18 + H]^+^. The following fragmentation pattern of fragment *m*/*z* 379.3359 was the breakage of the side chain to produce the fragment *m*/*z* 253.1952 [M – 144 + H]^+^, and the further breakage of the d-ring to produce the fragment *m*/*z* 213.1635 [M – 184 + H]^+^. Through another way, the direct breakage of the side chain and further breakage of the d-ring of parent ion *m*/*z* 397.3460 could yield the fragment *m*/*z* 271.2058 [M – 126 + H]^+^ and *m*/*z* 231.1739 [M – 166 + H]^+^. Likewise, by this means, the other 11 sterols in *S. crispa* were identified as strophasterol C (peak 44) [80], 9,11-dehydroergosterolperoxide (peak 76) [81], ganodermaside D (peak 79), ergosta-1,5,7,9(11),22-pentaen-3-one (peak 80) [82], 3-*O*-β-d-glucopyranosyl ergosterol peroxide (peak 81) [9], (5α,6α)-epoxy-ergosta-8(14),22-diene-3β,7β-diol (peak 85) [6], 3β,5α,9α-trihydroxyergosta-7,22-dien-6-one (peak 89) [83], ergone (peak 90) [84], 3-hydroxyergosta-5,8,22-trien-7-one (peak 95) [85], ergosterol peroxide (peak 96) [9], and ergosta-5,8,22-triene-3,11-dione (peak 100) [86], respectively. Among them, strophasterol C, 9,11-dehydroergosterolperoxide, ganodermaside D, ergosta-1,5,7,9(11),22-pentaen-3-one, 3β,5α,9α-trihydroxyergosta-7,22-dien-6-one, ergone, 3-hydroxyergosta-5,8,22-trien-7-one, and ergosta-5,8,22-triene-3,11-dione were components identified from the genus of *Sparassis* for the first time.

#### 2.3.5. Structural Characterization and Identification of Phthalides and Others Types of Compounds

A total of 13 phthalides were identified from the extracts of *S. crispa*. As depicted in Figure S5, in positive ion mode, the fragmentation process of protonated molecular ion *m*/*z* 233.1170 [M + H]^+^ of fraxinellone (**5**, peak 61) [87] started from the intramolecular dehydration to form the fragment ion *m*/*z* 215.1067 [M – 18 + H]^+^, and then its secondary dehydration to form the fragment *m*/*z* 197.0961 [M – 36 + H]^+^. The decarbonylation of fragment *m*/*z* 215.1067 led to the production of fragment *m*/*z* 187.1117 [M – 46 + H]^+^, and then its secondary decarbonylation led to the fragment *m*/*z* 159.1169 [M – 74 + H]^+^. Under the higher collision energy, the fragment *m*/*z* 187.1117 could dehydrate to form the fragment *m*/*z* 169.1012 [M – 64 + H]^+^. The fragment *m*/*z* 95.0497 [M – 138 + H]^+^ was derived from the breakage of the A-ring. In this way, the other 12 phthalides were identified as sparalide B (peak 11) [6], hanabiratakelide C (peak 14) [7], hanabiratakelide B (peak 15) [7], sparalide C (peak 17) [6], hanabiratakelide A (peak 19) [6,7], 5-hydroxy-7-methoxyphthalide (peak 20) [6], sparalide A (peak 21) [6], 6-hydroxy-5,7-dimethoxyphthalide (peak 23) [7], 4-hydroxy-5,7-dimethoxy-1-isobenzofuranone (peak 24) [7], 5-methoxy-7-hydroxyphthalide (peak 29) [6], meconin (peak 31) [87], and 5,7-dimethoxyphthalide (peak 32) [88], respectively. Among them, two compounds, meconin and fraxinellone, were identified from the genus of *Sparassis* for the first time.

Except those, there were also other types of compounds identified. As a typical representative, the MS/MS fragmentation of mannitol (peak 2, **6**) [89] was firstly investigated. Its protonated molecular ion was *m*/*z* 183.0863 [M + H]^+^ in positive ESI mode, and its main fragmentation pattern was continuous dehydration, resulting in the production of stepdown fragment ions, such as *m*/*z* 165.0760 [M – 18 + H]^+^, 147.0652 [M – 36 + H]^+^, 129.0548 [M – 54 + H]^+^, and 111.0444 [M – 72 + H]^+^. For the other types of compounds with various structures, the fragmentation patterns included but were not confined to dehydration, decarbonylation, decarboxylation, ring-opening and rearrangement. Finally, in total 21 other compounds were identified or tentatively identified, including ribono-1,4-lactone (peak 5) [56], citric acid monomethyl ester (peak 6) [90], crispacolide (peak 10) [91], methyl-2, 4-dihydroxy-6-methylbenzoate (peak 18) [10], sparoside A (peak 27) [6], methyl 2,4-dihydroxy-3-methoxy-6-methylbenzoate (peak 28) [6], methyl dihydroxymethoxy-methylbenzoate (peak 30) [10], sparassol (peak 33) [10], glucitol (peak 34) [10], dulcitol (peak 35) [57], xanthoangelol (peak 42) [92], 4-hydroxyderricin (peak 51) [92], butylated hydroxyanisole (peak 53) [93], armillarin (peak 62) [94], nicandrose E (peak 64) [95], 1,3-di(isobutoxycarbonyl)-2,4,4-trimethylpentane (peak 65) [96], 3-hydroxy-2-[[(9Z)-1-oxo-9-hexadecen-1-yl]oxy]propyl β-d-galactopyranoside (peak 70) [97], 1-(9-octadecenoate)-β-d-glucopyranose (peak 72) [98], 2,3-bis(4-hydroxy-3-methoxybenzyl)butane-1,4-diylditetradecanoate (peak 75) [99], linolenic acid ethyl ester (peak 84) [100], and 2,2′-methylenebis(4-methyl-6-tert butylphenol) (peak 92) [101]. Among them, 14 compounds including mannitol, ribono-1,4-lactone, citric acid monomethyl ester, glucitol, dulcitol, butylated hydroxyanisole, armillarin, nicandrose E, 1,3-di(isobutoxycarbonyl)-2,4,4-trimethylpentane, 3-hydroxy-2-[[(9Z)-1-oxo-9-hexadecen-1-yl]oxy]propyl β-d-galactopyranoside, 1-(9-octadecenoate)-β-d-glucopyranose, 2,3-bis(4-hydroxy-3-methoxybenzyl)butane-1,4-diylditetradecanoate, linolenic acid ethyl ester, and 2,2′-methylenebis(4-methyl-6-tert butylphenol) were identified from the genus of *Sparassis* for the first time.

### 2.4. In Silico Prediction of Potential SFKs Inhibitors

Eleven compounds showed strong affinity for Hck (Table S2a), 21 compounds for Lyn (Table S2b), 10 compounds for Fyn (Table S2c), and 6 compounds for Syk (Table S2d). All these compounds probably could be SFKs inhibitors. In particular, both 10-hydroxy-8,12-octadecadienoic acid and 9-hydroxy-10,14-octadecadien-12-ynoic acid had strong affinity for the above four enzymes, while both 2-amino-1,3-hexadecanediol and hurghamide D had strong affinity for Hck, Lyn, and Syk, which indicated that they were likely to be multi-targeted SFKs inhibitors.

**Figure 2:**
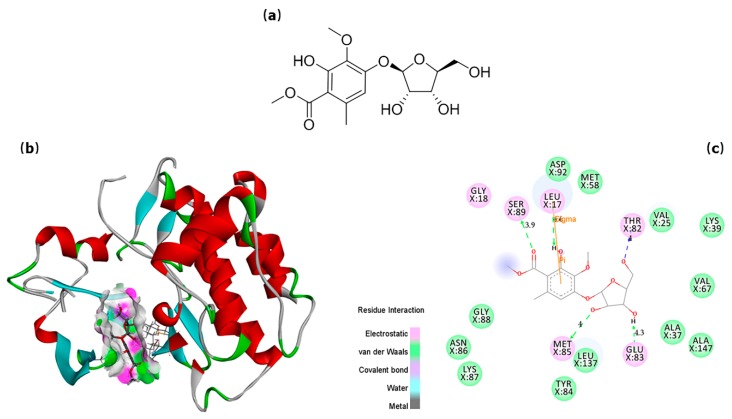
Chemical structure of the best-fit candidate and its match mode with targeted receptor. (**a**) Chemical structure of the best-fit candidate sparoside A. (**b**) Optimal confirmation of sparoside A bonded into the protein active site of Fyn. (**c**) Interaction pattern of sparoside A with amino acid residues of Fyn (the balls represented residues within active site, red capital letters represented interacting atoms, and dotted arrows represented hydrogen bonds).

To further understand the interaction between drugs and targeted enzymes, the confirmations of small molecules bonded into the “active cavity” of proteins and their interaction patterns with amino acid residues of protein was simulated via Discovery Studio Visualizer 4.0. Taking the active compound sparoside A (Figure 2a) as an example, as shown in Figure 2b, the small molecule was “curling up” in the protein active site of Fyn, which was unambiguously presented in the graphical molecular ribbon model. It was found to form five hydrogen bonds with five residues in chain X, including with Leu17 (4.6 Å) on hydroxyl, with Ser89 (3.9 Å) on carboxyl, with Thr82 (4.0 Å), Glu83 (4.3 Å), and Met85 (4.0 Å) on glucosyl group (Figure 2c). The other intermolecular interactions were also depicted, which were included but not confined to electrostatic force and van der Waals force. It was that all these interactions contributed to the high anti-inflammatory activity of sparoside A.

### 2.5. Anti-inflammatory Activity Confirmation of Two Predicted Components

**Figure 3:**
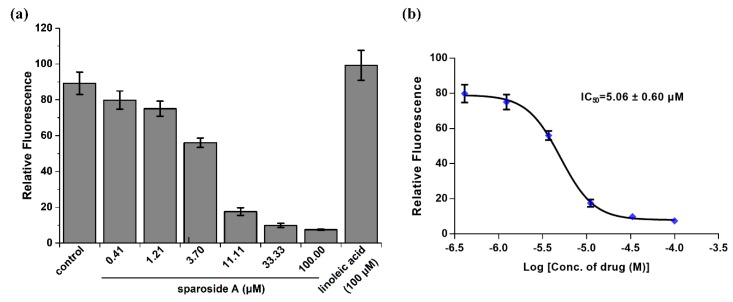
Anti-inflammatory activity evaluation of two predicted components via intracellular calcium mobilization assay. (**a**) Ca^2+^ mobilization in Furo-2AM loaded RBL-2H3 cells was compared when treating with different concentrations of sparoside A and 100 μM linoleic acid. 0.25% dimethylsulfoxide (DMSO) was used as the vehicle control. (**b**) Sparoside A dose-dependently decreased intracellular Ca^2+^ mobilization, and the IC_50_ value was calculated from the logarithmic concentration-response curve. All error bars indicate the standard deviation in six replicates.

To investigate the anti-inflammatory effects of those predicted hits, Ca^2+^ mobilization in Furo-2AM loaded RBL-2H3 cells was measured. As shown in Figure 3a, compared to the control vehicle, sparoside A (100 μM) significantly decreased intracellular Ca^2+^ concentration, while linoleic acid slightly increased it. This result suggested that sparoside A was a positive inhibitor of SFKs, considering by combining the aforementioned docking results. But linoleic acid was probably an agonist of SFKs, although it owned strong affinity with SFKs as the inhibitors. The inhibitors and agonists couldn’t be distinguished by virtual screening. In the next experiment, it was found that sparoside A dose-dependently decreased intracellular Ca^2+^ mobilization, and its IC_50_ value was 5.06 ± 0.60 μM in six replicates (Figure 3b).

## 3. Materials and Methods

### 3.1. Reagents and Materials

*S. crispa* was cultured by Shanxi Agricultural University, China, and its pileus was collected for the current investigation. Voucher specimens were preserved at the authors’ laboratory.

The reference compound riboflavin (**1**) with purity ≥98% was purchased from Shanghai Standard Technology Co., Ltd. (Shanghai, China); citric acid (**2**), ergosterol (**4**), fraxinellone (**5**) and mannitol (**6**) all with purity ≥98% were from Shanghai Yuanye Biological Technology Co., Ltd. (Shanghai, China); sparoside A (**7**) and linoleic acid (**8**) both with purity ≥98% were from Quality Phytochemicals (East Brunswick, NJ, USA); and ainsliatone A (**3**) with purity ≥95% was isolated from other herbs in our laboratory and unequivocally identified by NMR and high-resolution MS.

Acetonitrile, methanol, formic acid (all MS grade) and dimethylsulfoxide (DMSO, HPLC grade) were purchased from Sigma-Aldrich (St. Louis, MO, USA). Dulbecco’s modified Eagle’s medium (DMEM), fetal bovine serums (FBS), phosphate buffer solution (PBS, 100 mM, pH 7.4), and antibiotics were purchased from Thermo Fisher Scientific (Waltham, MA, USA). Flua-2AM dye was from Molecular Probes (Grand Island, NY, USA). The ultra-pure water was prepared with the Millipore-Q water purification system (Bedford, MA, USA).

### 3.2. Sample Preparation

After accurately weighed and grounded, 1.0 g air-dried pileus of *S. crispa* was extracted with 20 mL methanol in a 50 mL erlenmeyer flask by ultrasonic extraction for 30 min. After cooling down, the lost volume of methanol was complemented. Then 5.0 mg of six reference compounds were dissolved into 5 mL methanol to get six standard solutions, respectively. Finally, the above herb extracts solution and all standard solutions were filtered through a 0.22 μm membrane as the samples.

### 3.3. UPLC Separation

UPLC separation was carried out on a Thermo Vanquish Flex Binary RSLC platform (Thermo Fisher Scientific, Waltham, MA, USA) equipped with a diode array detector (DAD). The chromatographic column used was a Thermo Accucore aQ C_18_ (150 × 2.1 mm, 2.6 μm; Thermo Fisher Scientific, Waltham, MA, USA), which conducted in 40 °C. The mobile phase was composed of 0.1% formic acid aqueous solution (A) and acetonitrile (B), and the gradient elution program was as follows: 5%–100% B at 0–20 min; 100% B at 20–23 min. The flow rate was constant at 0.4 mL/min. The injection volume was set at 2 μL.

### 3.4. Orbitrap MS Analysis and Data Processing

The Q Exactive Plus mass spectrometer (Thermo Fisher Scientific, Waltham, MA, USA) was coupled to the UPLC by a HESI interface. The specific parameters were set as abovementioned. The mass spectrometer calibration was conducted before each experiment. In the MS/MS experiments, data-dependent scanning was adopted to trigger the second stage fragmentation, which was to select the strongest four parent ions in each scanning point of MS^1^ as targeted precursor ions for the further fragmentation. The dynamic exclusion function was utilized to prevent the repetitive ion scans and save the analysis time. The software Xcalibur 4.1 (Thermo Fisher Scientific, Waltham, MA, USA) and Compound Discoverer 2.1 (Thermo Fisher Scientific, Waltham, MA, USA) loaded with OTCML database 1.0 (Thermo Fisher Scientific, Waltham, MA, USA) were employed to process the UPLC-MS data. To ensure the reliability of the identification results, those peaks with intensity over 10^5^ in TIC were selected for identification. The formulas of all parent and fragment ions in selected peaks were generated according to their accurate mass using a formula predictor. The maximal mass accuracy error was confined to ±3 ppm. Considering the possible elemental compositions of existed compounds in *S. crispa*, the number of four types of atoms were limited as follows: C ≤ 50, H ≤ 100, O ≤ 20, and N ≤ 10.

### 3.5. Virtual Screening for Potential SFKs Inhibitors

To predict the SFKs inhibitors in *S. crispa*, molecular modelling and virtual screening based on docking were performed using Surflex-Dock GeomX (SFXC) program [102] interfaced with SYBYL-X 2.1.1 (Tripos, USA) on Dell Precision T5500 workstation. SFXC is a fast and automated docking program that considers ligand conformational flexibility by an incremental fragment placing technique. It was used to dock the small molecules into the active site of the protein and fished out the best-fit compounds. The 3D coordinates of the active site of four kinds of SFKs were taken from the reported X-ray crystal structure of the protein catalytic core in complex with a cocrystallized natural ligand from RCSB Protein Databank (https://www.rcsb.org). The corresponding PDB codes of Hck, Lyn, Fyn and Syk were 5H0B [103], 5XY1 [104], 2DQ7 [105], and 6HM7 [106], respectively. They were picked as the queries to screen the self-built 3D chemical database including 110 identified compounds derived from *S. crispa*. As the positive control, the natural ligands were also docked via the identical procedure. The “protomol” for docking was defined as all amino acids within 6.5 Å proximity of the natural ligands, and the other parameters were set as default. Finally, the Total scores were calculated to denote the matching degree between the conformers of each compound and the four enzymes, along with other reference fit values including Crash, Polar, Similarity, D score, PMF score, G score, Chem score, and C score; namely, a higher Total score indicated a better match. The docking results were visualized and analyzed with Discovery Studio Visualizer 4.0 (Accelrys, USA).

### 3.6. Anti-inflammatory Activity Evaluation by Intracellular Calcium Mobilization Assay

Extracellular Ca^2+^ influx was an essential process in activating mast cells and other related immune cells to induce allergic reactions [107]. Thus, the intracellular calcium mobilization assay was employed to evaluate the anti-inflammatory activity of those hits in virtual screening. Due to the unavailability of pure substances, only linoleic acid and sparoside A were applied in this research. The two kind of drugs were dissolved in DMSO, and 0.25% DMSO were used as the control vehicle. The final concentration of DMSO in each well did not exceed 0.25% for all of the tested drugs. Rat basophilic leukemia (RBL)-2H3 cells [108] were obtained from the American Type Culture Collection (ATCC, Manassas, VA, USA) and cultured at 37 °C in DMEM supplemented with 10% FBS and antibiotics (100 U/mL penicillin, 100 μg/mL streptomycin) in a 5% CO_2_ incubator. The intracellular Ca^2+^ level ([Ca^2+^]_i_) was measured using Fura-2AM loading by monitoring the fluorescence intensity. First, cells were pretreated with drug solutions or vehicle for 1 h at 37 °C, and thereafter, washed with Ringer’s Solution (155 mM NaCl, 4.5 mM KCl, 2 mM MgCl_2_, 10 mM dextrose, 5 mM HEPES, pH 7.4), supplemented with 1 mM CaCl_2_. Then they were loaded with 1 μM Fura-2AM at a concentration of 10^7^ cells/mL for 1 h in the dark. At last, the cells were washed, resuspended in Ca^2+^ supplemented Ringer’s Solution, and the [Ca^2+^]_i_ of 5 × 10^5^ cells was monitored on Quanta master Spectrofluorometer (Photon Technology International, Birmingham, NJ, USA) at room temperature. Ca^2+^ mobilization was expressed as the ratio (Relative Fluorescence, RF) of Fura-2AM fluorescence at 510 nM caused by the two excitation wavelengths (340 nm/380 nm). The IC_50_ value was calculated by RF. Each datum represents the mean ± standard deviation in six replicates.

### 3.7. Data Process and Analysis

All data acquired were processed using one-way analysis of variance (ANOVA), followed by Student’s *t*-test to find the differences between group means in GraphPad Prism v7.0 (GraphPad Software, La Jolla, CA, USA). The level of significance was set at less than 5% (*p* < 0.05).

## 4. Conclusions

In conclusion, an UPLC coupled with Orbitrap MS method was firstly developed and applied for rapid separation and characterization of chemical components in *S. crispa*. Based on reference compounds, optimized UPLC and MS conditions, and systematic fragment ions-based identification strategy, a total of 110 compounds of interest were detected and identified or tentatively identified. The MS/MS fragmentation patterns of all the characterized compounds in positive/negative ion modes were also explored. Furthermore, it was found that in UPLC eluted with gradient acetonitrile and 0.1% formic acid aqueous solution, the retention time of different types of chemical constituents in *S. crispa* was roughly as the following order: organic acids < phthalides < sterols/sesquiterpenes, and the occurrence of alkaloids covered the entire separation time. To our knowledge, this is the first study to systematically establish the chemical composition profile of *S. crispa* by UPLC-MS, and the method presented here has been demonstrated as an effective pathway for the analysis of the components in a complex sample from natural resource. In addition, in order to explore the inhibitory effects of *S. crispa* on AR, virtual screening was conducted to predict the inhibitors of SFKs based on the self-built chemical substance database. Two available compounds sparoside A and linoleic acid were applied in intracellular calcium mobilization assay to evaluate their anti-inflammatory activity. Finally, it was confirmed directly for the first time that sparoside A showed obvious anti-inflammatory activity, which could dose-dependently decrease intracellular Ca^2+^ mobilization with IC_50_ value 5.06 ± 0.60 μM. This result was an important improvement over the previous data from Bang et al. [12]. As to whether sparoside A could treat AR, further pharmacological studies are needed in the future. This study could provide essential reference for the medicinal and edible research and development on this kind of mushroom.

## Figures and Tables

**Table 1:** The ultra-high performance liquid chromatography-mass spectrometer (UPLC-MS) data of six representative reference compounds in *Sparassis crispa*.

Category	Compound Name	t_R_ (min)	Formula	Exact Mass	Adduct Ion *m*/*z*	Mass Error (ppm)	Fragment Ion *m*/*z*
alkaloid	riboflavin (**1**) ^a^	3.97	C_17_H_20_O_6_N_4_	376.1383	377.1459 [M + H]^+^	0.820	359(35.6) ^b^, 341(20.3), 243(100), 99(16.7)
organic acid	citric acid (**2**)	0.80	C_6_H_8_O_7_	192.0270	−191.0195 [M − H]^-^	−0.920	173(41.9), 129(35.0), 111(100), 85(31.8)
sesquiterpene	ainsliatone A (**3**)	4.57	C_14_H_18_O_4_	250.1200	251.1278 [M + H]^+^ 273.1095 [M + Na]^+^	0.257 −0.953	233(32.2), 215(65.0), 205(48.5), 197(7.3), 187(100),169(28.1), 159(20.5), 145(33.9)
sterol	ergosterol (**4**)	18.48	C_28_H_44_O	396.3392	397.3463 [M + H]^-^	−0.610	379(100), 271(58.7), 253(31.2), 231(40.0), 213(53.1)
phthalide	fraxinellone (**5**)	11.34	C_14_H_16_O_3_	232.1094	233.1171 [M + H]^+^	−0.390	215(100), 197(9.9), 187(73.5), 169(13.9), 159(18.0), 95(22.3)
other	mannitol (**6**)	0.76	C_6_H_14_O_6_	182.0790	183.0864 [M + H]^+^ 205.0683 [M + Na]^+^	0.521 0.198	165(70.0), 147(44.1), 129(32.2), 111(100)

**^a^** The bracketed bold figures shows the serial number of corresponding reference compounds. **^b^** The bracketed figures following *m/z* shows the relative abundance (%) of each fragment ion.

## Supplementary Materials

The following can be found at online. Table S1: All the identified components from *Sparassis crispa* extracts and their UPLC-MS/MS data; Table S2: The virtual screening results for self-built 3D chemical database including 110 compounds in *S. crispa* with four kinds of SFKs (Hck, Lyn, Fyn and Syk) as the receptors; Figure S1: MS spectra and proposed fragment ions of riboflavin (**1**) in positive ion mode; Figure S2: MS spectra and proposed fragment ions of citric acid (**2**) in negative ion mode; Figure S3: MS spectra and proposed fragment ions of ainsliatone A (**3**) in positive ion mode; Figure S4: MS spectra and proposed fragment ions of ergosterol (**4**) in positive ion mode; Figure S5: MS spectra and proposed fragment ions of fraxinellone (**5**) in positive ion mode; Figure S6: MS spectra and proposed fragment ions of mannitol (**6**) in positive ion mode; Figure S7: Comparison of total ion current chromatograms (TIC) of *S. crispa* extracted with different solvents in positive and negative ion modes; Figure S8: Chemical structures and available raw MS^2^ spectra of some components identified from *S. crispa.*

## Abbreviations

UPLC	ultra-high performance liquid chromatography
GC	gas chromatography
MS	mass spectrometer
CID	collision-induced dissociation
NCE	normalized collision energy
AR	allergic rhinitis
SFKs	Src family kinases
DMEM	Dulbecco’s modified Eagle’s medium
FBS	fetal bovine serums
PBS	phosphate buffer solution
DMSO	dimethylsulfoxide
