# Supplementary material for: Rapid Characterization of Chemical Components in Edible Mushroom Sparassis crispa by UPLC-Orbitrap MS Analysis and Potential Inhibitory Effects on Allergic Rhinitis
Source: Molecules. 2019 Aug 20;24(16):3014. doi: 10.3390/molecules24163014 (PMC6720900; doi:10.3390/molecules24163014)
Supplement: Supplementary file 1 [file molecules-24-03014-s001.pdf]

Table S1. All the identified components from *Sparassis crispa* extracts and their UPLC-MS data.

Peak No.	Retention time （min）	Positive ESI mode			Negative ESI mode			Exact mass	Formula	Identification	Confidence level [a]
		Adduct ion m/z	Mass error （ppm）	Fragment ion m/z	Adduct ion m/z	Mass error （ppm）	Fragment ion m/z				
1	0.71	175.1190 [M+H]$^+$	0.558	158, 130, 116	173.1043 [M-H]$^-$	-0.687	-	174.1117	$C_6H_{14}O_2N_4$	arginine	III
2	0.76	183.0864 [M+H]$^+$<br>205.0683 [M+Na]$^+$	0.521<br>0.198	165, 147, 129, 111	181.0716 [M-H]$^-$	-0.836	-	182.0790	$C_6H_{14}O_6$	mannitol (**6**) [b]	I
3	0.80	-	-	-	191.0195 [M-H]$^-$	-0.920	173, 129, 111, 85	192.0270	$C_6H_8O_7$	citric acid (**2**)	I
4	1.05	276.1554 [M+H]$^+$	0.010	159, 144, 131, 120, 116, 88	274.1406 [M-H]$^-$	-0.927	-	275.1481	$C_{11}H_{21}O_5N_3$	ethyl-L-glutaminyl-L-threonine	III
5	1.07	-	-	-	147.0297 [M-H]$^-$	-1.337	129, 115, 103	148.0372	$C_5H_8O_5$	ribono-1,4-lactone	III
6	1.31	207.0499 [M+H]$^+$	0.052	-	205.0352 [M-H]$^-$	-1.004	173, 155, 143, 111	206.0427	$C_7H_{10}O_7$	citric acid monomethyl ester	III
7	1.49	-	-	-	147.0661 [M-H]$^-$	-1.238	129, 101	148.0736	$C_6H_{12}O_4$	mevalonic acid	III
8	1.69	-	-	-	131.0348 [M-H]$^-$	-0.109	113	132.0423	$C_5H_8O_4$	methylsuccinic acid	III
9	2.44	-	-	-	145.0505 [M-H]$^-$	0.911	127, 101	146.0579	$C_6H_{10}O_4$	3-methylglutaric acid	III
10	2.61	185.0809 [M+H]$^+$<br>207.0629 [M+Na]$^+$	0.457<br>0.338	141, 109	-	-	-	184.0736	$C_9H_{12}O_4$	crispacolide	III
11	2.64	-	-	-	211.0247 [M-H]$^-$	-0.717	193, 165	212.0321	$C_9H_8O_6$	sparalide B	III
12	2.65	298.0967 [M+H]$^+$	-0.425	280, 262, 136	296.0820 [M-H]$^-$	-1.058	-	297.0896	$C_{11}H_{15}O_3N_5S$	5′-deoxy-5′-methylthioadenosine	III
13	2.74	229.1548 [M+H]$^+$	-0.572	-	227.1400 [M-H]$^-$	-0.510	183, 168, 152, 139, 124, 114	228.1474	$C_{11}H_{20}O_3N_2$	leucylproline	III
14	2.90	-	-	-	211.0247 [M-H]$^-$	-0.290	193, 165	212.0321	$C_9H_8O_6$	hanabiratakelide C	III
15	3.00	-	-	-	195.0298 [M-H]$^-$	-0.649	165	196.0372	$C_9H_8O_5$	hanabiratakelide B	III

Peak No.	Retention time （min）	Positive ESI mode			Negative ESI mode			Exact mass	Formula	Identification	Confidence level [a]
		Adduct ion m/z	Mass error （ppm）	Fragment ion m/z	Adduct ion m/z	Mass error （ppm）	Fragment ion m/z				
16	3.30	-	-	-	188.0927 $[M-H]^-$	-0.698	144, 116	189.1001	$C_8H_{15}O_4N$	2-aminooctanedioic acid	III
17	3.33	-	-	-	195.0298 $[M-H]^-$<br>391.0669 $[2M-H]^-$	-0.547<br>-0.358	177, 149	196.0372	$C_9H_8O_5$	sparalide C	III
18	3.48	183.0652 $[M+H]^+$	0.244	-	181.0506 $[M-H]^-$	-0.453	123, 95, 67	182.0579	$C_9H_{10}O_4$	methyl-2, 4-dihydroxy-6-methylbenzoate	III
19	3.54	-	-	-	195.0298 $[M-H]^-$	-0.495	177, 149	196.0372	$C_9H_8O_5$	hanabiratakelide A	III
20	3.70	181.0497 $[M+H]^+$	0.634	-	179.0349 $[M-H]^-$	-0.235	149	180.0423	$C_9H_8O_4$	5-hydroxy-7-methoxyphthalide	III
21	3.84	-	-	-	311.0773 $[M-H]^-$	0.191	293, 275, 245, 179, 149, 97	312.0854	$C_{14}H_{16}O_8$	sparalide A	III
22	3.97	377.1459 $[M+H]^+$	0.820	359, 341, 243, 99	375.1310 $[M-H]^-$	0.006	-	376.1383	$C_{17}H_{20}O_6N_4$	riboflavin (**1**)	I
23	4.08	211.0602 $[M+H]^+$<br>233.0421 $[M+Na]^+$	0.616<br>0.281	193, 165	209.0456 $[M-H]^-$	0.303	191, 163	210.0528	$C_{10}H_{10}O_5$	6-hydroxy-5,7-dimethoxyphthalide	III
24	4.18	211.0601 $[M+H]^+$<br>233.0421 $[M+Na]^+$	0.001<br>0.109	193, 165	209.0455 $[M-H]^-$	0.016	191, 163	210.0528	$C_{10}H_{10}O_5$	4-hydroxy-5,7-dimethoxy-1-isobenzofuranone	III
25	4.57	251.1278 $[M+H]^+$<br>273.1095 $[M+Na]^+$	0.257<br>-0.953	233, 215, 205, 197， 187， 169， 159， 145	-	-	-	250.1200	$C_{14}H_{18}O_4$	ainsliatone A (**3**)	I
26	4.67	-	-	-	173.0817 $[M-H]^-$	1.341	129, 111	174.0892	$C_8H_{14}O_4$	suberic acid	III
27	4.75	345.1181 $[M+H]^+$	0.352	-	343.1033 $[M-H]^-$	-0.453	285, 257, 195, 167, 137, 109	344.1107	$C_{15}H_{20}O_9$	sparoside A	III

Peak No.	Retention time （min）	Positive ESI mode			Negative ESI mode			Exact mass	Formula	Identification	Confidence level [a]
		Adduct ion m/z	Mass error （ppm）	Fragment ion m/z	Adduct ion m/z	Mass error （ppm）	Fragment ion m/z				
28	5.18	213.0756 $[M+H]^+$	-0.657	185, 157, 155, 127, 99	211.0611 $[M-H]^-$	-0.553	-	212.0685	$C_{10}H_{12}O_5$	methyl 2,4-dihydroxy-3-methoxy-6-methylbenzoate	III
29	5.35	181.0495 $[M+H]^+$	-0.471	151	179.0348 $[M-H]^-$	-0.570	149	180.0423	$C_9H_8O_4$	5-methoxy-7-hydroxyphthalide	III
30	5.41	213.0757 $[M+H]^+$	-0.328	155, 127	211.0612 $[M-H]^-$	0.158	-	212.0685	$C_{10}H_{12}O_5$	methyl dihydroxymethoxy-methylbenzoate	III
31	5.69	195.0652 $[M+H]^+$	0.075	165, 135	-	-	-	194.0579	$C_{10}H_{10}O_4$	meconin	III
32	5.70	195.0651 $[M+H]^+$	-0.232	165, 135	-	-	-	194.0579	$C_{10}H_{10}O_4$	5,7-dimethoxyphthalide	III
33	6.35	197.0809 $[M+H]^+$	0.125	169, 139,111	-	-	-	196.0736	$C_{10}H_{12}O_4$	sparassol	III
34	6.42	-	-	-	181.0717 $[M-H]^-$	-0.394	163, 149, 131, 119, 101, 89, 71, 59	182.0790	$C_6H_{14}O_6$	glucitol	III
35	6.72	-	-	-	181.0716 $[M-H]^-$		163, 131, 119, 101	182.0790	$C_6H_{14}O_6$	dulcitol	III
36	6.83	218.2115 $[M+H]^+$	0.157	200, 174, 156, 130, 106	-	-	-	217.2042	$C_{12}H_{27}O_2N$	2-amino-1,3-dodecanediol	III
37	6.85	655.3084 $[M+H]^+$	-0.295	343, 315, 244, 226, 112	653.2943 $[M-H]^-$	0.398	-	654.3013	$C_{32}H_{42}O_9N_6$	stellarin C	III
38	6.97	-	-	-	201.1131 $[M-H]^-$	-0.658	183, 139	202.1205	$C_{10}H_{18}O_4$	3-tert-butyladipic acid	III
39	7.15	262.2377 $[M+H]^+$	0.113	244, 226, 136, 118, 100	-	-	-	261.2304	$C_{14}H_{31}O_3N$	2-amino-1,3,4-tetradecanetriol	III

Peak No.	Retention time （min）	Positive ESI mode			Negative ESI mode			Exact mass	Formula	Identification	Confidence level [a]
		Adduct ion m/z	Mass error （ppm）	Fragment ion m/z	Adduct ion m/z	Mass error （ppm）	Fragment ion m/z				
40	7.25	376.2483 $[M+H]^+$	0.146	360, 332, 316, 288	374.2336 $[M-H]^-$	-0.112	-	375.2410	$C_{22}H_{33}O_4N$	tuberostemonine	III
41	7.60	436.2693 $[M+H]^+$	-0.170	-	434.2552 $[M-H]^-$	0.803	416, 398, 206, 193	435.2621	$C_{24}H_{37}O_6N$	salternamide D	III
42	7.69	393.2068 $[M+H]^+$	1.994	365, 337, 309	-	-	-	392.1988	$C_{25}H_{28}O_4$	xanthoangelol	III
43	7.87	293.2110 $[M+H]^+$	-0.516	275, 233, 177, 151, 99, 71	-	-	-	292.2083	$C_{18}H_{28}O_3$	9-hydroxy-10,14-octadecadien-12-ynoic acid	III
44	8.09	459.3104 $[M+H]^+$	-0.307	441, 423, 277, 259, 241	-	-	-	458.3032	$C_{28}H_{42}O_5$	strophasterol C	III
45	8.20	556.3052 $[M+H]^+$	-1.024	496, 434, 394, 374, 324	554.2906 $[M-H]^-$	0.884	-	555.2985	$C_{35}H_{41}O_5N$	3-(acetyloxy)-17-(benzoyloxy)- 7-[O-(phenylmethyl)oxime] androst-5-en-7-one	III
46	8.33	267.1590 $[M+H]^+$	-0.433	-	265.1444 $[M-H]^-$	--0.575	247	266.1518	$C_{15}H_{22}O_4$	clitocybulol C	III
47	8.45	295.2266 $[M+H]^+$	-0.411	277, 221	293.2120 $[M-H]^-$	-0.710	275	294.2195	$C_{18}H_{30}O_3$	3β-hydroxy-11,12-O-isopropylidenedrimene	III
48	8.48	-	-	-	329.2331 $[M-H]^-$	-0.751	229, 211, 171, 139	330.2406	$C_{18}H_{34}O_5$	9,12,13-trihydroxy-15-octadecenoic acid	III
49	8.61	277.2161 $[M+H]^+$	-0.529	-	275.2016 $[M-H]^-$	-0.194	231	276.2089	$C_{18}H_{28}O_2$	moroctic acid	III
50	8.77	332.1847 $[M+H]^+$	-2.754	274, 264, 206	-	-	-	331.1784	$C_{19}H_{25}O_4N$	antrodin D	III
51	8.79	339.1597 $[M+H]^+$	1.811	311, 283, 251	-	-	-	358.1518	$C_{21}H_{22}O_4$	4-hydroxyderricin	III

Peak No.	Retention time（min）	Positive ESI mode			Negative ESI mode			Exact mass	Formula	Identification	Confidence level [a]
		Adduct ion m/z	Mass error（ppm）	Fragment ion m/z	Adduct ion m/z	Mass error（ppm）	Fragment ion m/z				
52	8.99	526.3309 $[M+H]^+$	-1.217	-	524.3160 $[M-H]^-$	1.706	508, 506, 492, 490, 474, 360, 257	525.3243	$C_{35}H_{43}O_3N$	1-(18-benzamido-3β-hydroxy-5α-androstan-17β-yl)-3-phenyl-2-propen-1-one	III
53	9.11	181.1223 $[M+H]^+$	-0.035	153, 149, 121	-	-	-	180.1150	$C_{11}H_{16}O_2$	butylated hydroxyanisole	III
54	9.31	353.2298 $[M+H]^+$	0.665	337, 335, 321, 303	351.2155 $[M-H]^-$	1.446	-	352.2223	$C_{16}H_{28}O_3N_6$	3-cyclohexene-1-butyraldehyde-3-hydroxy-α-isopropyl-1-methyl-2-oxo-disemicarbazone	III
55	9.69	313.2374 $[M+H]^+$	0.204	-	311.2227 $[M-H]^-$	-0.394	293, 249, 171	312.2301	$C_{18}H_{32}O_4$	5,8-dihydroxy-9,12-octadecadienoic acid	III
56	9.86	-	-	-	267.1599 $[M-H]^-$	-1.057	249, 223	268.1675	$C_{15}H_{24}O_4$	ustusol B	III
57	10.34	-	-	-	265.1447 $[M-H]^-$	0.632	244	266.1518	$C_{15}H_{22}O_4$	3-hydroxy-(4,2-hydroxy-6-methyl)-2 heptanyl benzoic acid	III
58	10.56	311.2216 $[M+H]^+$	-0.405	293, 275, 257, 223	309.2070 $[M-H]^-$	-0.429	291, 273, 209, 185, 99	310.2144	$C_{18}H_{30}O_4$	porrigenic acid	III

Peak No.	Retention time（min）	Positive ESI mode			Negative ESI mode			Exact mass	Formula	Identification	Confidence level [a]
		Adduct ion m/z	Mass error（ppm）	Fragment ion m/z	Adduct ion m/z	Mass error（ppm）	Fragment ion m/z				
59	10.63	330.1707 [M+H]$^+$	2.015	272, 262, 204	-	-	-	329.1627	$C_{19}H_{23}O_4N$	antrodin C	III
60	10.70	293.2109 [M+H]$^+$	-0.755	275, 229, 177, 161, 151, 133	-	-	-	292.2038	$C_{18}H_{28}O_3$	12-oxo-phytodienoic acid	III
61	11.34	233.1171 [M+H]$^+$	-0.390	215, 197, 187, 169, 159, 95	-	-	-	232.1094	$C_{14}H_{16}O_3$	fraxinellone (**5**)	I
62	11.36	-	-	-	415.2112 [M-H]$^-$	-0.735	119	414.2042	$C_{24}H_{30}O_6$	armillarin	III
63	12.12	337.2736 [M+H]$^+$	-0.479	293, 275	-	-	-	336.2664	$C_{21}H_{36}O_3$	3-methyl-5-pentyl-2-furanundecanoic acid	III
64	12.51	-	-	-	649.3440 [M-H]$^-$	1.551	562, 341, 323, 279, 87	650.3514	$C_{31}H_{54}O_{14}$	nicandrose E	III
65	12.78	315.2530 [M+H]$^+$	-0.083	-	313.2383 [M-H]$^-$	-0.296	295, 277, 201, 183	314.2457	$C_{18}H_{34}O_4$	1,3-di(isobutoxycarbonyl)-2,4,4-trimethylpentane	III
66	12.92	318.3002 [M+H]$^+$	-0.316	300, 282, 264, 68	-	-	-	317.2930	$C_{18}H_{39}O_3N$	2-amino-1,3,4-trihydroxyoctadecane	III
67	13.13	-	-	-	295.2275 [M-H]$^-$	-1.111	277, 251, 228, 183	296.2351	$C_{18}H_{32}O_3$	10-hydroxy-8,12-octadecadienoic acid	III
68	13.31	279.2317 [M+H]$^+$	-0.561	235	-	-	-	278.2246	$C_{18}H_{30}O_2$	α-eleostearic acid	III
69	13.49	-	-	-	295.2276 [M-H]$^-$	-0.908	277, 251, 228, 183	296.2351	$C_{18}H_{32}O_3$	8-oxo-9-octadecenoic acid	III

Peak No.	Retention time （min）	Positive ESI mode			Negative ESI mode			Exact mass	Formula	Identification	Confidence level [a]
		Adduct ion m/z	Mass error （ppm）	Fragment ion m/z	Adduct ion m/z	Mass error （ppm）	Fragment ion m/z				
70	14.05	-	-	-	489.3066 $[M-H]^-$	-0.667	279	490.3142	$C_{25}H_{46}O_9$	3-hydroxy-2-[[(9Z)-1-oxo-9-hexadecen-1-yl]oxy]propyl β-D-galactopyranoside	III
71	14.20	-	-	-	297.2433 $[M-H]^-$	-0.869	279, 251, 207, 155	298.2508	$C_{18}H_{34}O_3$	6,7-epoxystearic acid	III
72	14.33	445.3159 $[M+H]^+$	-0.068	409, 343, 285, 267, 187	443.3013 $[M-H]^-$	-0.354	-	444.3087	$C_{24}H_{44}O_7$	1-(9-octadecenoate)-β-D-glucopyranose	III
73	14.36	295.2267 $[M+H]^+$	-0.241	255, 129, 101	-	-	-	294.2195	$C_{18}H_{30}O_3$	9-oxo-octadecadienoic acid	III
74	14.50	-	-	-	503.3012 $[M-H]^-$	-0.371	383, 361, 339, 299, 279	504.3087	$C_{29}H_{44}O_7$	2,3-seco-2,3-dicarboxyplatanic acid	III
75	15.09	782.5689 $[M+H]^+$	-0.346	727, 573, 555, 499, 363, 345, 335, 327, 317, 307, 299, 289, 271	-	-	-	782.5697	$C_{48}H_{78}O_8$	2,3-bis(4-hydroxy-3-methoxybenzyl)butane-1,4-diylditetradecanoate	III
76	15.46	427.3205 $[M+H]^+$	-0.332	409, 301, 283, 261, 243	425.3063 $[M-H]^-$	0.222	-	426.3134	$C_{28}H_{42}O_3$	9,11-dehydroergosterolp	III

Peak No.	Retention time （min）	Positive ESI mode			Negative ESI mode			Exact mass	Formula	Identification	Confidence level [a]
		Adduct ion m/z	Mass error （ppm）	Fragment ion m/z	Adduct ion m/z	Mass error （ppm）	Fragment ion m/z				
										eroxide	
77	15.47	274.2739 $[M+H]^+$	-0.714	256, 237, 84	-	-	-	273.2668	$C_{16}H_{35}O_2N$	2-amino-1,3-hexadecanediol	III
78	15.59	483.2868 $[M+H]^+$	0.671	465, 422, 406	-	-	-	482.2781	$C_{28}H_{38}O_5N_2$	stachybotrin G	III
79	15.68	409.3100 $[M+H]^+$	-0.261	391, 283, 265, 243, 225	-	-	-	408.3028	$C_{28}H_{40}O_2$	ganodermaside D	III
80	15.69	391.2994 $[M+H]^+$	-0.262	265, 225	-	-	-	390.2923	$C_{28}H_{38}O$	ergosta-1,5,7,9(11),22-pentaen-3-one	III
81	15.83	591.3885 $[M+H]^+$<br>613.3708 $[M+Na]^+$	-1.006<br>-0.391	411, 465, 425, 285, 245	-	-	-	590.3819	$C_{34}H_{54}O_8$	3-O-β-D-glucopyranosyl ergosterol peroxide	III
82	16.14	340.2846 $[M+H]^+$	-0.207	298, 280, 238, 112	-	-	-	339.2773	$C_{20}H_{37}O_3N$	2-methyl-6-(11-oxododecyl)piperidin-3-yl acetate	III
83	16.50	307.2631 $[M+H]^+$	-0.218	263	-	-	-	306.2559	$C_{20}H_{34}O_2$	diroleuton	III
84	16.51	307.2631 $[M+H]^+$	-0.185	292, 261, 219, 191, 173, 149, 123, 109	-	-	-	306.2559	$C_{20}H_{34}O_2$	linolenic acid ethyl ester	III
85	16.63	429.3362 $[M+H]^+$	-0.260	411, 393, 375, 303, 285, 267, 263, 249, 245, 227, 209	-	-	-	428.3290	$C_{28}H_{44}O_3$	(5α,6α)-epoxy-ergosta-8(14),22-diene-3β,7β-diol	III

Peak No.	Retention time （min）	Positive ESI mode Adduct ion m/z	Mass error （ppm）	Fragment ion m/z	Negative ESI mode Adduct ion m/z	Mass error （ppm）	Fragment ion m/z	Exact mass	Formula	Identification	Confidence level [a]
86	16.64	368.3160 $[M+H]^+$	0.243	326, 308, 266, 112	366.3012 $[M-H]^-$	-0.348	-	367.3086	$C_{22}H_{41}O_3N$	2-methyl-6-(13-oxotetradecyl)piperidin-3-yl acetate	III
87	16.67	326.3053 $[M+H]^+$	-0.171	309, 121	-	-	-	325.2981	$C_{20}H_{39}O_2N$	oleoylethanolamide	III
88	16.85	380.3159 $[M+H]^+$	-0.159	350, 322, 267, 239	378.3013 $[M-H]^-$	-0.178	-	379.3086	$C_{23}H_{41}O_3N$	hurghamide D	III
89	16.90	445.3315 $[M+H]^+$	0.480	427, 409, 391, 319, 301, 283, 279, 261, 265, 243, 225	-	-	-	444.3240	$C_{28}H_{44}O_4$	3β,5α,9α-trihydroxyergosta-7,22-dien-6-one	III
90	16.99	393.3150 $[M+H]^+$	-0.489	267, 227	-	-	-	392.3079	$C_{28}H_{40}O$	ergone	III
91	17.02	318.3002 $[M+H]^+$	-0.222	300, 282, 100	-	-	-	317.2930	$C_{18}H_{39}O_3N$	2-amino-1,3,4-octadecanetriol	III
92	17.03	-	-	-	339.2327 $[M-H]^-$	-0.747	163	340.2402	$C_{23}H_{32}O_2$	2,2'-methylenebis(4-methyl-6-tert butylphenol)	III
93	17.56	-	-	-	279.2326 $[M-H]^-$	-1.266	261	280.2402	$C_{18}H_{32}O_2$	linoleic acid	III
94	17.92	282.2790 $[M+H]^+$	0.501	265, 247, 191, 177, 163, 135, 121, 111	280.2650 $[M-H]^-$	0.462	-	281.2719	$C_{18}H_{35}NO$	oleamide	III
95	18.05	411.3255 $[M+H]^+$	-0.698	393, 285, 253, 245, 213,	409.3112 $[M-H]^-$	0.016	-	410.3185	$C_{28}H_{42}O_2$	3-hydroxyergosta-5,8,22-trien-7-one	III
96	18.24	429.3363 $[M+H]^+$ 451.3179 $[M+Na]^+$	0.019 -0.745	411, 393, 375, 303, 287, 285,	-	-	-	428.3290	$C_{28}H_{44}O_3$	ergosterol peroxide	III

Peak No.	Retention time （min）	Positive ESI mode			Negative ESI mode			Exact mass	Formula	Identification	Confidence level [a]
		Adduct ion m/z	Mass error （ppm）	Fragment ion m/z	Adduct ion m/z	Mass error （ppm）	Fragment ion m/z				
				269, 263, 249, 245, 209							
97	18.37	256.2635 $[M+H]^+$	0.043	241, 213	-	-	-	255.2562	$C_{16}H_{33}ON$	hexadecanamide	III
98	18.48	397.3463 $[M+H]^-$	-0.610	379, 271, 253, 231, 213	369.3393 [M+H]-	-0.474	-	396.3392	$C_{28}H_{44}O$	ergosterol (**4**)	Ⅰ
99	18.58	384.3259 $[M+H]^+$	-0.420	366, 261, 257	382.3114 [M-H]-	-0.523	-	383.3188	$C_{26}H_{41}ON$	veracintine	III
100	19.04	409.3099 $[M+H]^+$	-0.555	283, 243	407.2952 $[M-H]^-$	-0.354	-	408.3028	$C_{28}H_{40}O_2$	ergosta-5,8,22-triene-3,11-dione	III
101	19.52	312.3259 $[M+H]^+$	-0.517	265	-	-	-	311.3188	$C_{20}H_{41}ON$	2-nonadecananone-O-methyloxime	III
102	19.67	386.3416 $[M+H]^+$	-0.496	370, 274, 258	-	-	-	385.3345	$C_{26}H_{43}ON$	4-azacholest-5-en-3-one	III
103	19.69	360.3260 $[M+H]^+$	-0.282	344, 213, 134	358.3116 [M-H]-	0.279	-	359.3188	$C_{24}H_{41}ON$	N-(2-phenylethyl)hexadecanamide	III
104	20.47	284.2946 $[M+H]^+$	-0.673	268, 241	-	-	-	283.2875	$C_{18}H_{37}ON$	stearamide	III
105	21.45	710.5559 $[M+H]^+$	-0.893	532, 484, 338, 294, 276	-	-	-	709.5493	$C_{41}H_{75}O_8N$	thraustochytroside A	III
106	21.54	-	-	-	772.5578 $[M-H]^-$	-0.305	564, 546, 420, 292	773.5653	$C_{42}H_{79}O_{11}N$	rhizoleucinoside	III
107	21.92	338.3417 $[M+H]^+$	-1.182	321, 303, 247, 163, 149, 135, 111	-	-	-	337.3345	$C_{22}H_{43}ON$	erucamide	III
108	21.96	579.4978 $[M+H]^+$	-0.866	-	577.4836 $[M-H]^-$	-0.292	377, 313, 295, 281	578.4910	$C_{36}H_{66}O_5$	1-(hydroxymethyl)-2[(1-oxohexadecyl)oxy]	III

Peak No.	Retention time （min）	Positive ESI mode			Negative ESI mode			Exact mass	Formula	Identification	Confidence level [a]
		Adduct ion m/z	Mass error （ppm）	Fragment ion m/z	Adduct ion m/z	Mass error （ppm）	Fragment ion m/z				
										ethyl-ester-9,12-heptadecadienoic acid	
109	24.22	241.2161_$[M+H]^+$	-0.401	-	239.2017 $[M-H]^-$	0.111	221, 203	240.2089	$C_{15}H_{28}O_2$	3-(10-hydroxy-10-methylethyl)-5,8a-dimethyldecahydro azulen-4-ol	III
110	24.84	241.2160_$[M+H]^+$	-0.650	-	239.2017 $[M-H]^-$	0.111	221, 203	240.2089 3.633	$C_{15}H_{28}O_2$	(5β, 6α)-6,11-dihydroxyeudesmane	III

[a)] The confidence level of identity of all the identified components were determined following the four levels defined by the Metabolomics Standards Initiative. Level Ⅰ: confidently identified compounds; Level Ⅱ: Putatively annotated compounds; Level Ⅲ: putatively annotated compound classes; Level Ⅳ: unknown compounds.

[b)] The bracketed bold figures showed the serial number of reference compounds.

Table S2. The virtual screening results for self-built 3D chemical database including 110 compounds in *S. crispa* with four kinds of SFKs (Hck, Lyn, Fyn and Syk) as the receptors.

(a) Hck

#	Compound name	Total score[a]	Crash	Polar	Similarity	D score	PMF score	G score	Chem score	C score
01	10-hydroxy-8,12-octadecadienoic acid	11.08	-0.92	4.70	0.46	-144.69	-16.55	-248.00	-25.14	4
02	salternamide D	10.94	-3.49	4.73	0.57	-187.83	-22.93	-365.29	-29.51	4
03	hurghamide D	10.30	-3.05	1.44	0.43	-164.76	4.96	-286.54	-24.63	4
04	2-amino-1,3-hexadecanediol	10.09	-2.01	4.46	0.38	-89.72	-16.98	-201.38	-26.19	5
05	2-methyl-6-(13-oxotetradecyl)piperidin-3-yl acetate	9.64	-1.68	0.90	0.47	-179.22	6.61	-355.41	-28.75	4
06	oleoyl ethanolamide	9.52	-2.05	1.89	0.37	-147.84	6.84	-257.37	-26.11	4
07	9-hydroxy-10,14-octadecadien-12-ynoic acid	9.13	-2.61	2.25	0.37	-152.93	-1.75	-243.76	-23.85	5
08	2,2'-methylenebis(4-methyl-6-tert butylphenol)	6.97	-1.73	0.93	0.27	8.91	-21.25	-272.47	-27.14	5
09	3-cyclohexene-1-butyraldehyde-3-hydroxy-α-isopropyl-1-methyl-2-oxo-disemicarbazone	6.84	-1.29	4.93	0.50	-236.90	-19.59	-428.79	-39.95	4
10	antrodin D	6.32	-3.63	0.40	0.55	-83.14	-18.19	-158.84	-4.05	4
11	5α,6α-epoxy-(22E,24R)-ergosta-8(14),22-diene-3β,7β-diol	6.27	-3.72	1.41	0.43	-147.91	-19.09	-319.39	-29.15	4
-	RK20449[b]	10.24	-1.14	2.11	0.71	-166.45	-81.46	-326.43	-34.69	5

(b) Lyn

#	Compound name	Total score[a]	Crash	Polar	Similarity	D score	PMF score	G score	Chem score	C score
01	thraustochytroside A	11.60	-5.30	2.07	0.44	-121.35	-16.73	-219.41	-21.40	4
02	2,3-bis(4-hydroxy-3-methoxybenzyl)butane-1,4-diylditetradecanoate	10.33	-3.57	1.06	0.41	-214.55	3.26	-372.06	-31.81	5
03	9-hydroxy-10,14-octadecadien-12-ynoic acid	9.72	-1.38	4.15	0.49	-139.90	3.70	-241.73	-23.60	4
04	1-(9-octadecenoate)-β-D-glucopyranose	9.43	-2.09	2.83	0.33	-154.63	-24.90	-308.10	-18.42	4
05	hurghamide D	9.30	-2.31	2.43	0.39	-133.97	-16.45	-234.93	-22.61	4
06	linolenic acid ethyl ester	9.25	-1.92	1.30	0.43	-60.32	-8.09	-150.06	-15.60	4
07	linoleic acid	8.41	-1.42	2.33	0.50	27.62	-20.11	-122.50	-17.89	5

#	Compound name	Total score[a]	Crash	Polar	Similarity	D score	PMF score	G score	Chem score	C score
08	salternamide D	8.24	-3.23	3.20	0.50	-161.63	-8.95	-272.14	-25.91	4
09	2-amino-1,3-hexadecanediol	8.22	-1.46	3.61	0.36	-94.74	-2.21	-220.80	-22.10	4
10	10-hydroxy-8,12-octadecadienoic acid	8.13	-1.30	3.27	0.58	-118.04	-5.55	-194.34	-19.87	5
11	2-amino-1,3,4-tetradecanetriol	7.90	-3.52	3.53	0.36	-132.73	-23.56	-223.69	-24.70	4
12	antrodin C	7.88	-1.92	2.01	0.30	-141.58	-4.70	-221.72	-23.68	4
13	sparoside A	7.68	-1.46	4.33	0.38	-130.97	-35.04	-202.86	-18.07	4
14	xanthoangelol	7.62	-3.37	1.97	0.49	-120.57	-7.98	-244.68	-27.34	4
15	antrodin D	7.59	-1.92	2.22	0.55	-74.60	-26.50	-139.27	-5.14	5
16	2-methyl-6-(13-oxotetradecyl)piperidin-3-yl acetate	7.55	-1.37	2.35	0.37	-151.12	-20.66	-281.24	-31.35	5
17	oleoyl ethanolamide	7.32	-1.39	2.75	0.34	-122.63	-27.37	-248.86	-26.24	4
18	9,11-dehydroergosterolperoxide	7.23	-1.52	1.16	0.36	-123.84	-8.61	-242.93	-26.40	4
19	5α,6α-epoxy-(22E,24R)-ergosta-8(14),22-diene-3β,7β-diol	6.68	-1.68	1.41	0.42	-126.19	-19.66	-265.73	-26.21	4
20	stachybotrin G	6.44	-3.23	2.06	0.50	-148.19	-22.74	-278.49	-26.62	4
21	ergosta-5,8,22-triene-3,11-dione	6.39	-2.29	0.35	0.39	-117.45	14.99	-254.63	-27.96	5
-	INNO-406[b]	8.52	-1.59	1.63	0.51	-150.29	-51.63	-252.22	-34.28	5

(c) Fyn

#	Compound name	Total score[a]	Crash	Polar	Similarity	D score	PMF score	G score	Chem score	C score
01	thraustochytroside A	11.28	-2.63	3.94	0.41	-140.27	-12.99	-259.44	-27.95	4
02	10-hydroxy-8,12-octadecadienoic acid	9.62	-0.95	3.27	0.55	-142.00	-12.25	-229.15	-23.84	5
03	antrodin D	8.61	-1.29	3.31	0.48	-71.56	-8.88	-91.25	-6.26	4
04	9-hydroxy-10,14-octadecadien-12-ynoic acid	8.54	-1.78	3.82	0.44	-143.64	-1.68	-241.54	-24.25	5
05	nicandrose E	8.52	-3.94	3.49	0.43	-80.82	-22.60	-125.52	-15.27	4
06	2-amino-1,3,4-tetradecanetriol	8.28	-1.74	2.91	0.33	-112.15	-9.15	-181.69	-16.81	4
07	linoleic acid	7.91	-4.05	1.25	0.29	15.56	-25.14	-117.63	-17.52	4
08	oleoyl ethanolamide	7.88	-1.46	2.18	0.39	-125.02	-12.83	-218.70	-19.90	4
09	sparalide A	7.51	-1.96	4.01	0.26	-87.78	-31.03	-227.42	-21.39	5

#	Compound name	Total score[a]	Crash	Polar	Similarity	D score	PMF score	G score	Chem score	C score
10	3-cyclohexene-1-butyraldehyde-3-hydroxy-α-isopropyl-1-methyl-2-oxo-disemicarbazone	6.42	-0.77	3.84	0.64	-191.24	-17.80	-331.39	-30.35	4
-	AZD0530[b]	6.56	-1.91	1.61	0.53	-269.05	-9.08	-257.50	-23.27	5

(d) Syk

#	Compound name	Total score [a]	Crash	Polar	Similarity	D score	PMF score	G score	Chem score	C score
01	10-hydroxy-8,12-octadecadienoic acid	8.73	-1.52	2.64	0.50	-133.84	-7.64	-235.06	-23.09	4
02	rhizoleucinoside	8.49	-6.29	4.23	0.40	-125.91	-17.80	-223.20	-24.29	5
03	hurghamide D	8.45	-2.01	0.96	0.50	-135.88	-28.62	-217.54	-22.80	5
04	3-cyclohexene-1-butyraldehyde-3-hydroxy-α-isopropyl-1-methyl-2-oxo-disemicarbazone	8.19	-0.86	4.67	0.54	-215.83	-66.85	-357.98	-36.63	4
05	9-hydroxy-10,14-octadecadien-12-ynoic acid	7.91	-0.90	3.99	0.54	-101.02	-1.03	-161.06	-17.98	4
06	2-amino-1,3-hexadecanediol	7.13	-1.57	3.32	0.40	-111.23	-35.13	-238.07	-23.52	4
-	RO9021[b]	6.32	-1.09	2.72	0.39	24559.12	-65.91	-213.14	-24.42	3

[a)] With the natural ligand of each protein as references, the RMSD was less than 2.0 for each match.

[b)] These compounds were known SFKs inhibitors, which were used as positive controls.

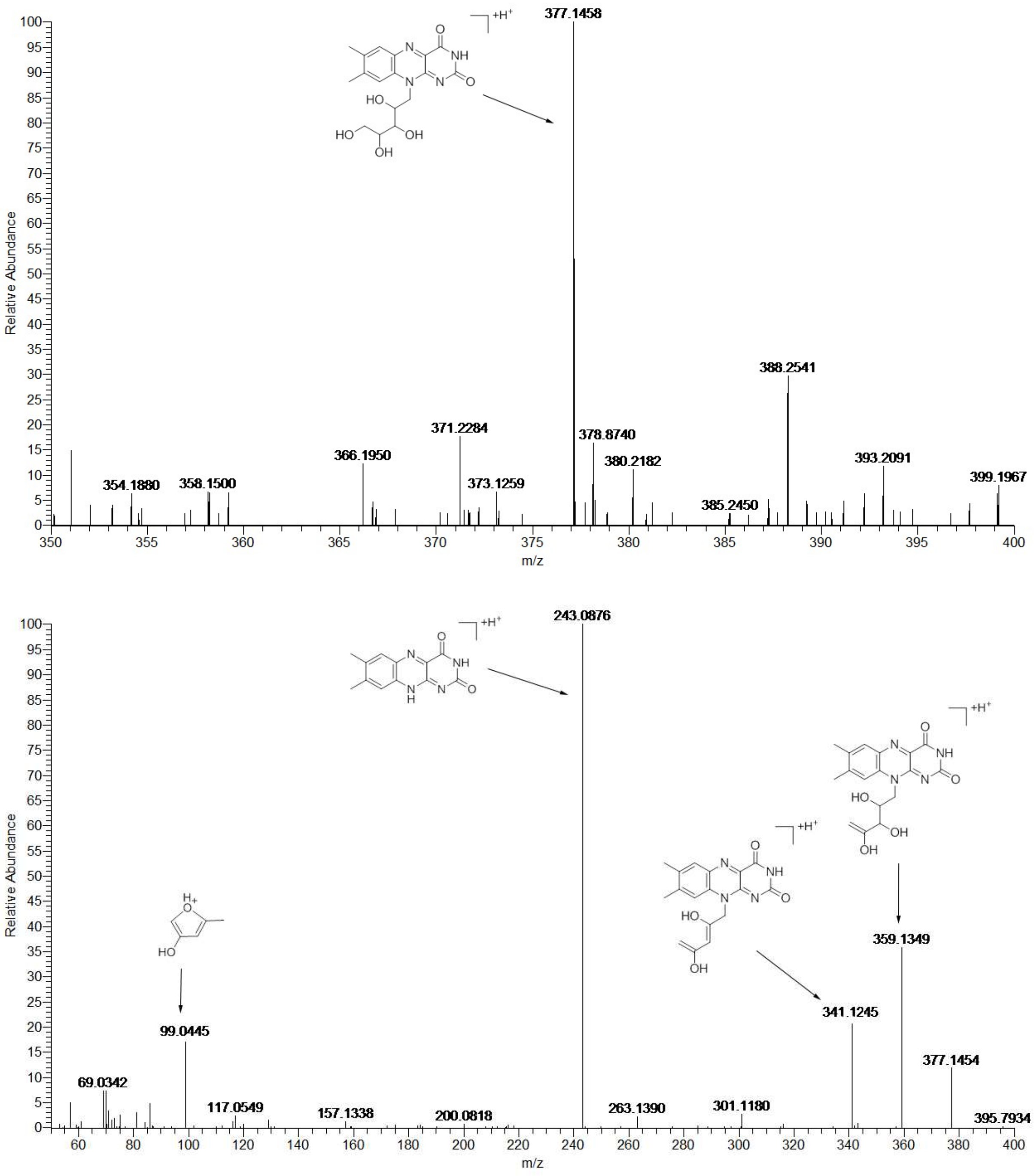

Figure S1. MS spectra and proposed fragment ions of riboflavin (**1**) in positive ion mode: (**a**) MS spectrum; (**b**) MS/MS spectrum (precursor ion was m/z 377).

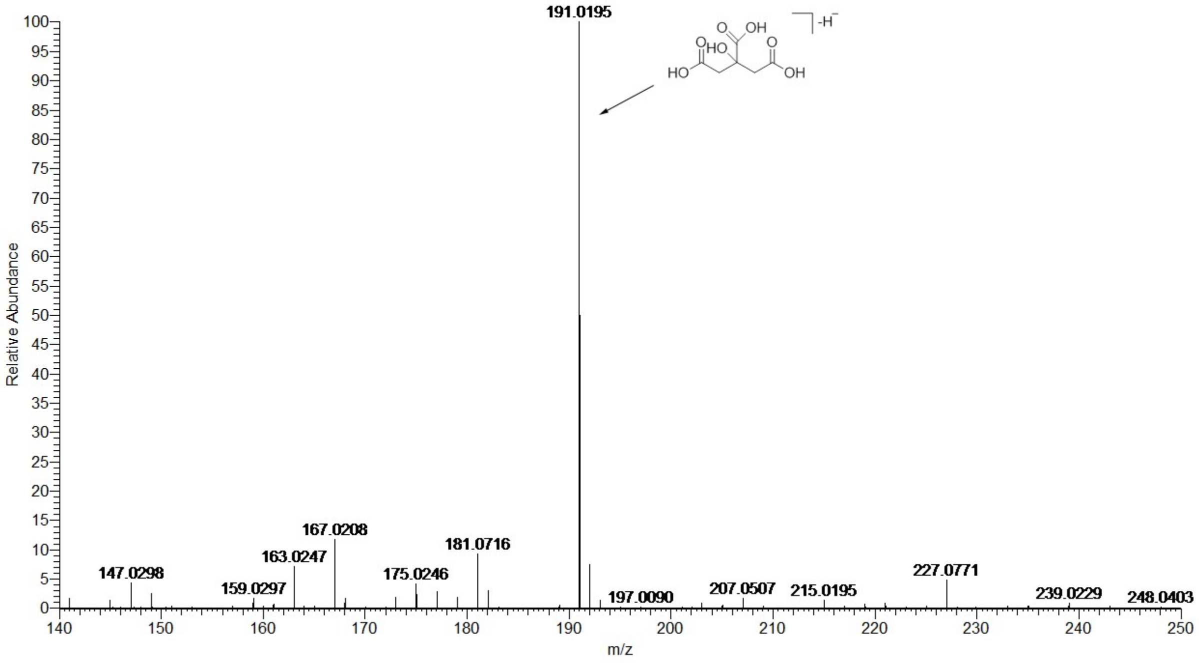

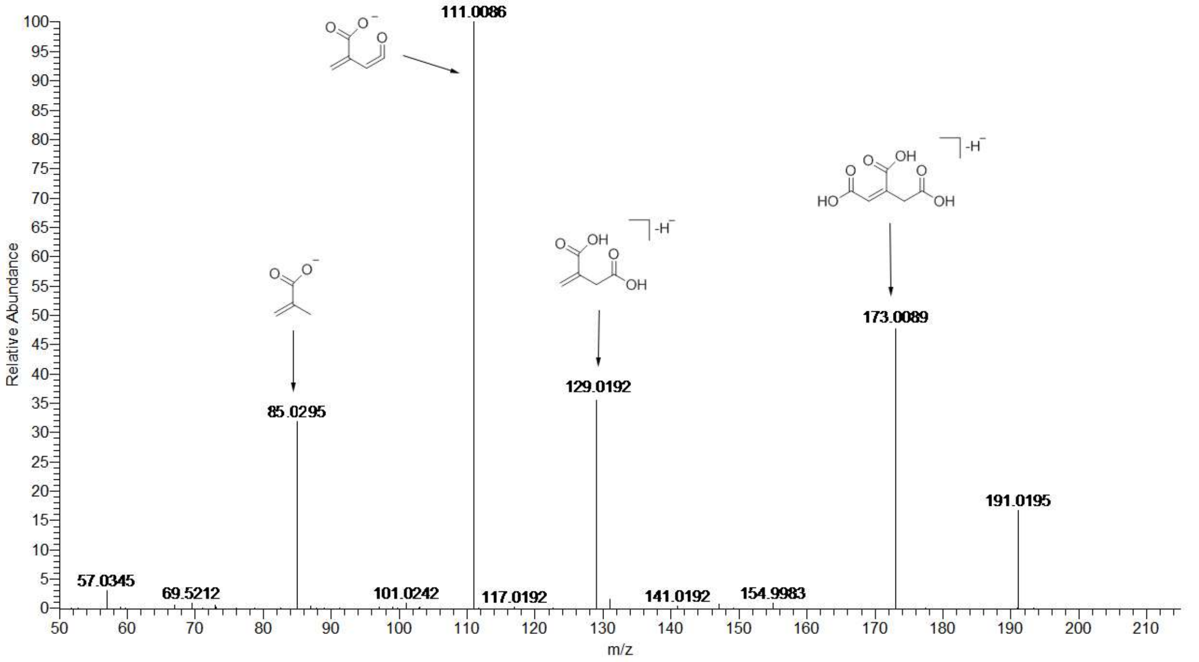

Figure S2. MS spectra and proposed fragment ions of citric acid (**2**) in negative ion mode: (**a**) MS spectrum; (**b**) MS/MS spectrum (precursor ion was m/z 191).

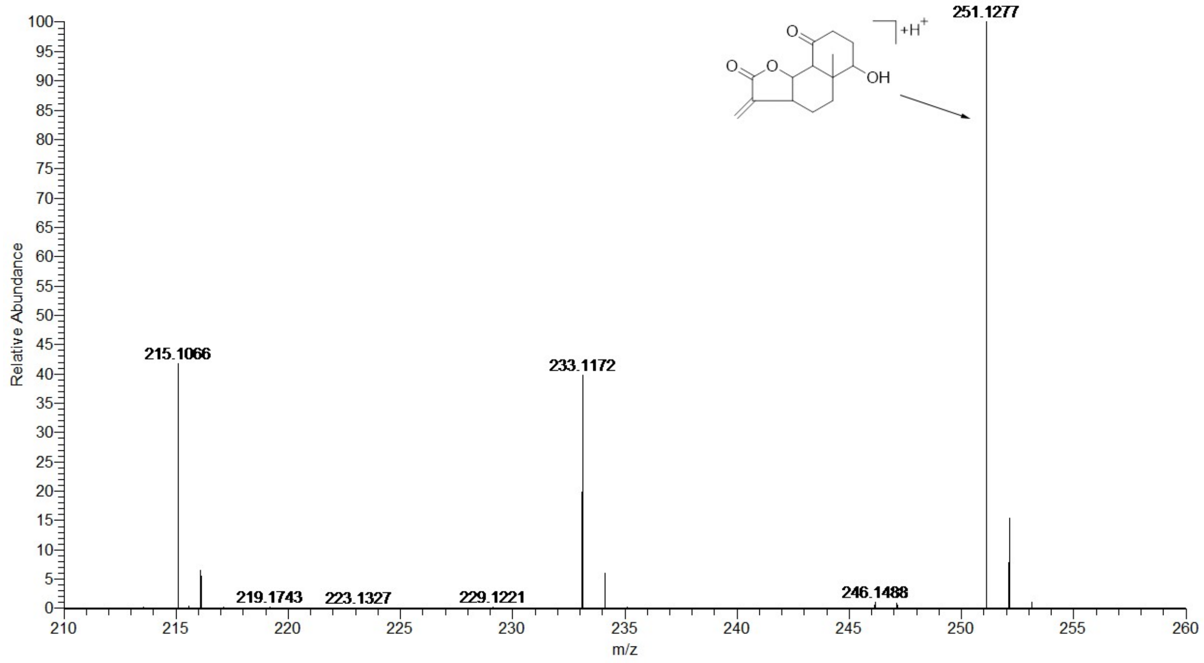

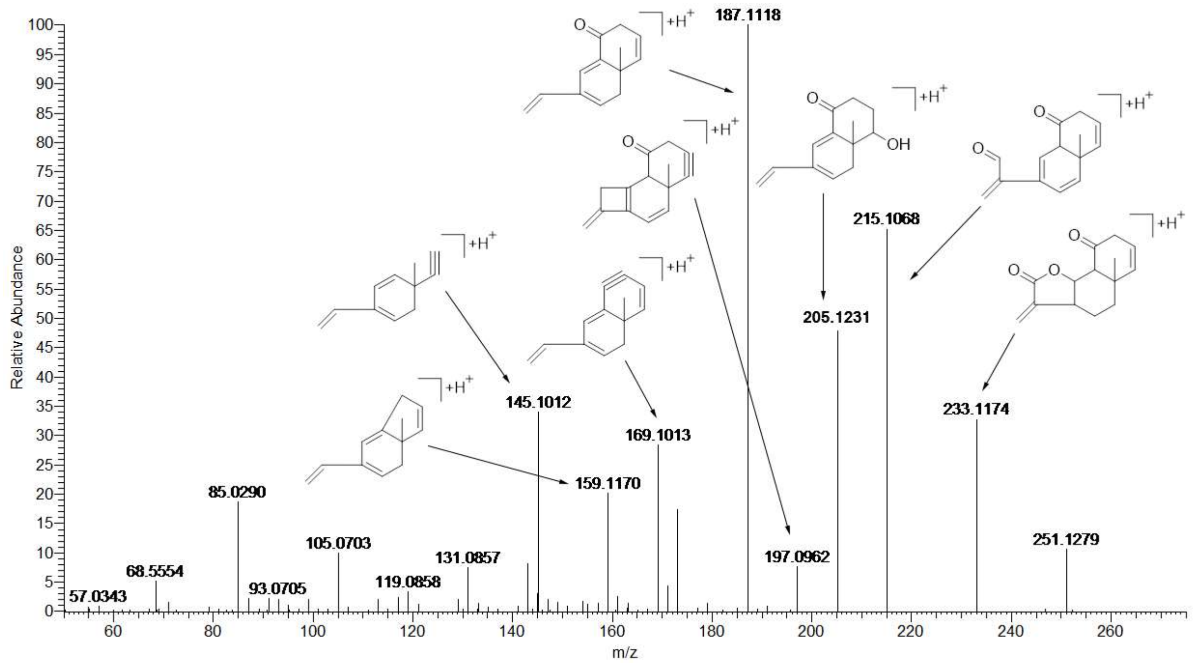

Figure S3. MS spectra and proposed fragment ions of ainsliatone A (**3**) in positive ion mode: (**a**) MS spectrum; (**b**) MS/MS spectrum (precursor ion was m/z 251).

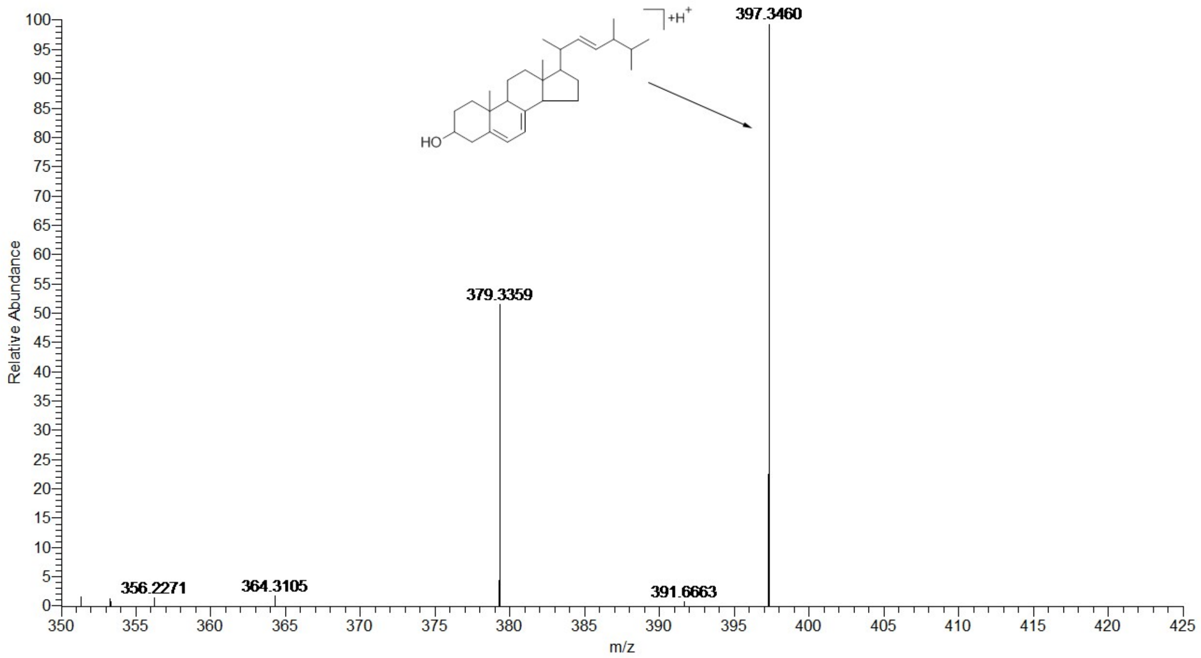

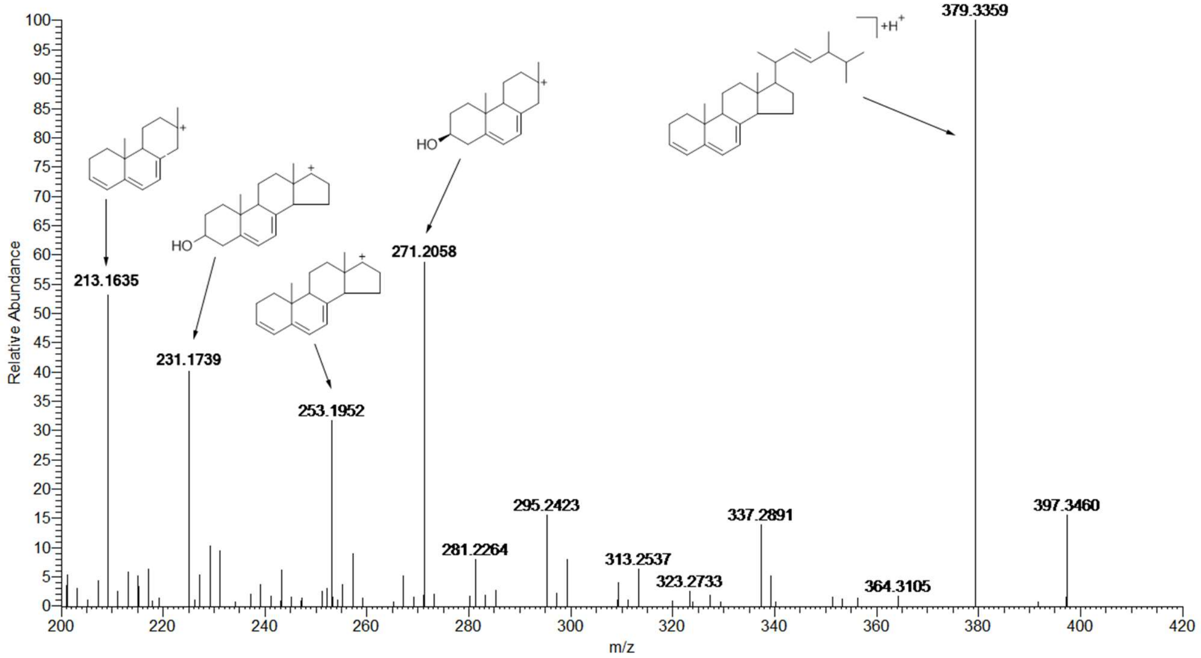

Figure S4. MS spectra and proposed fragment ions of ergosterol (**4**) in positive ion mode: (**a**) MS spectrum; (**b**) MS/MS spectrum (precursor ion was m/z 397).

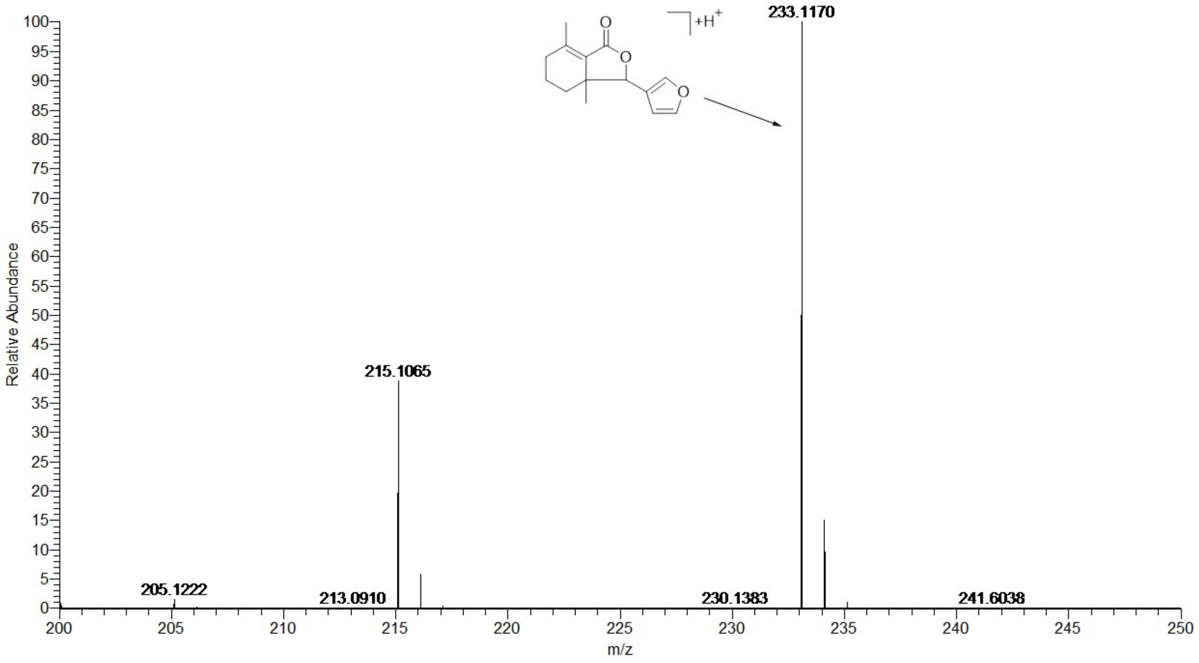

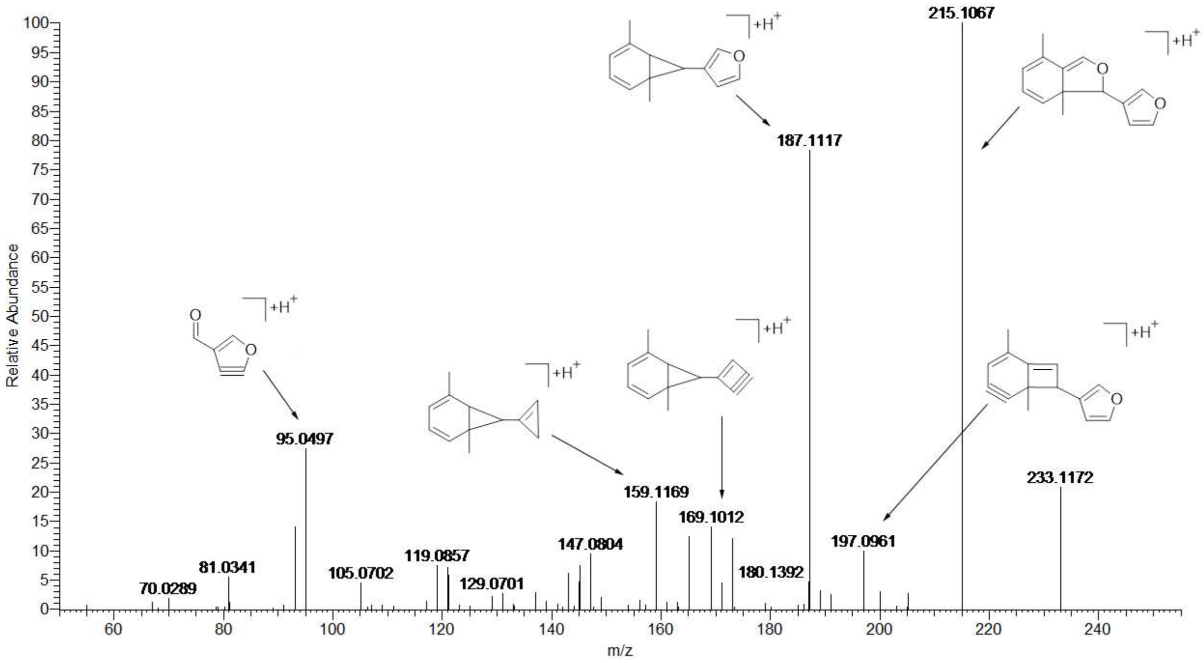

Figure S5. MS spectra and proposed fragment ions of fraxinellone (**5**) in positive ion mode: (**a**) MS spectrum; (**b**) MS/MS spectrum (precursor ion was m/z 233).

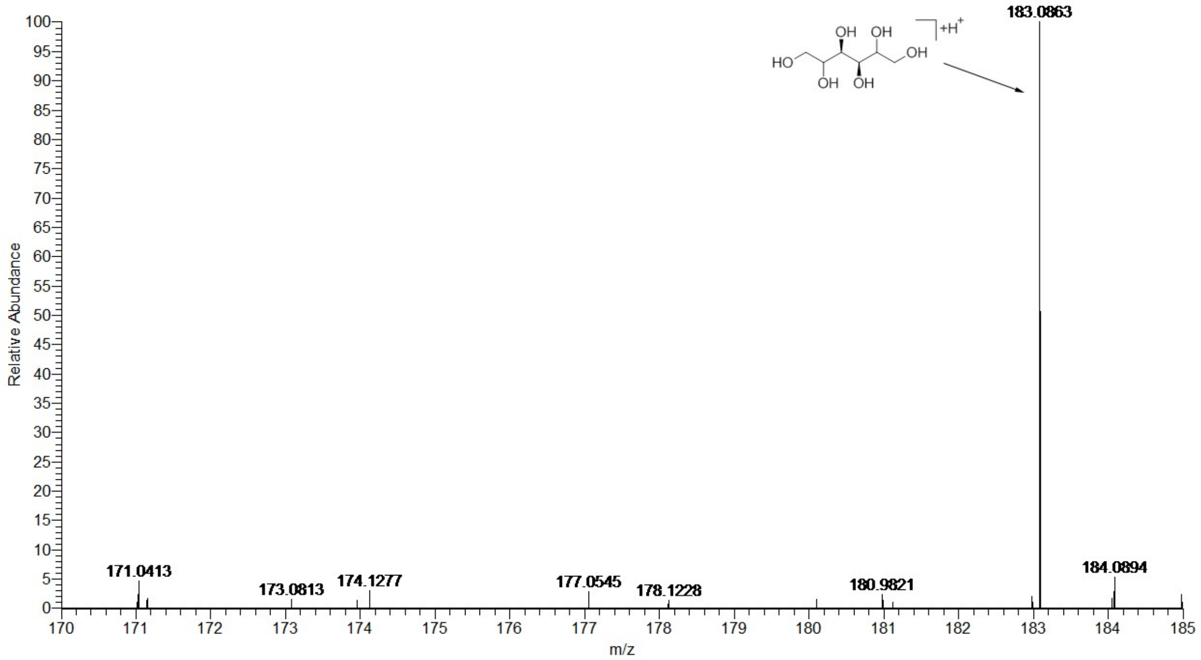

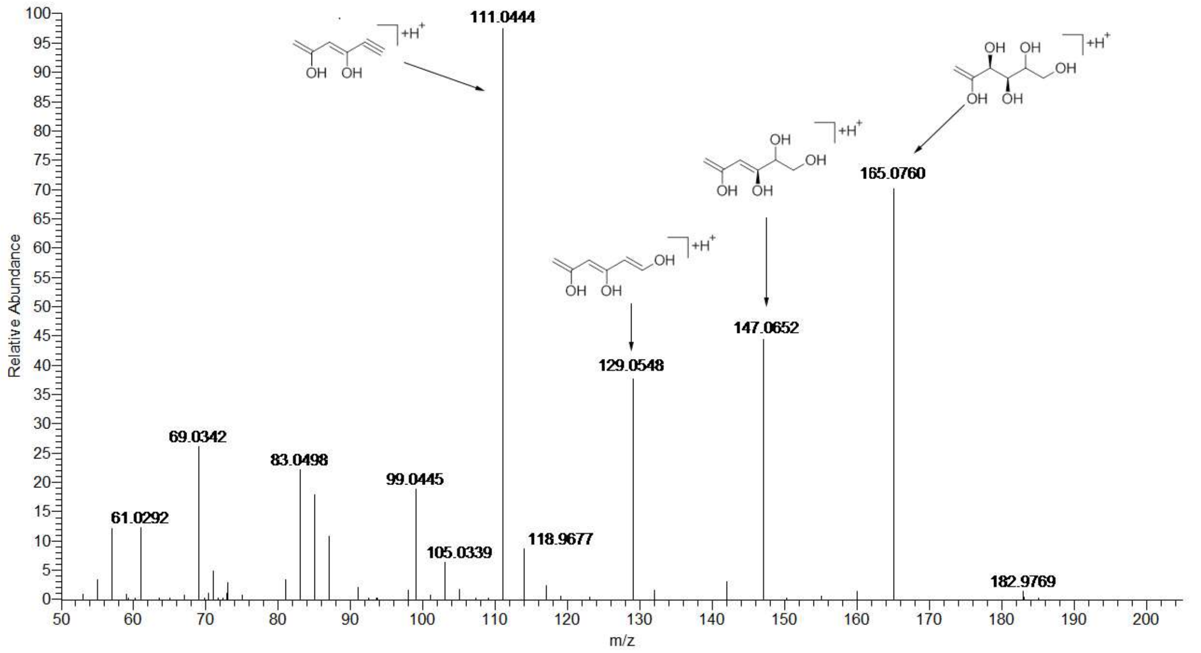

Figure S6. MS spectra and proposed fragment ions of mannitol (**6**) in positive ion mode: (**a**) MS spectrum; (**b**) MS/MS spectrum (precursor ion was m/z 183)

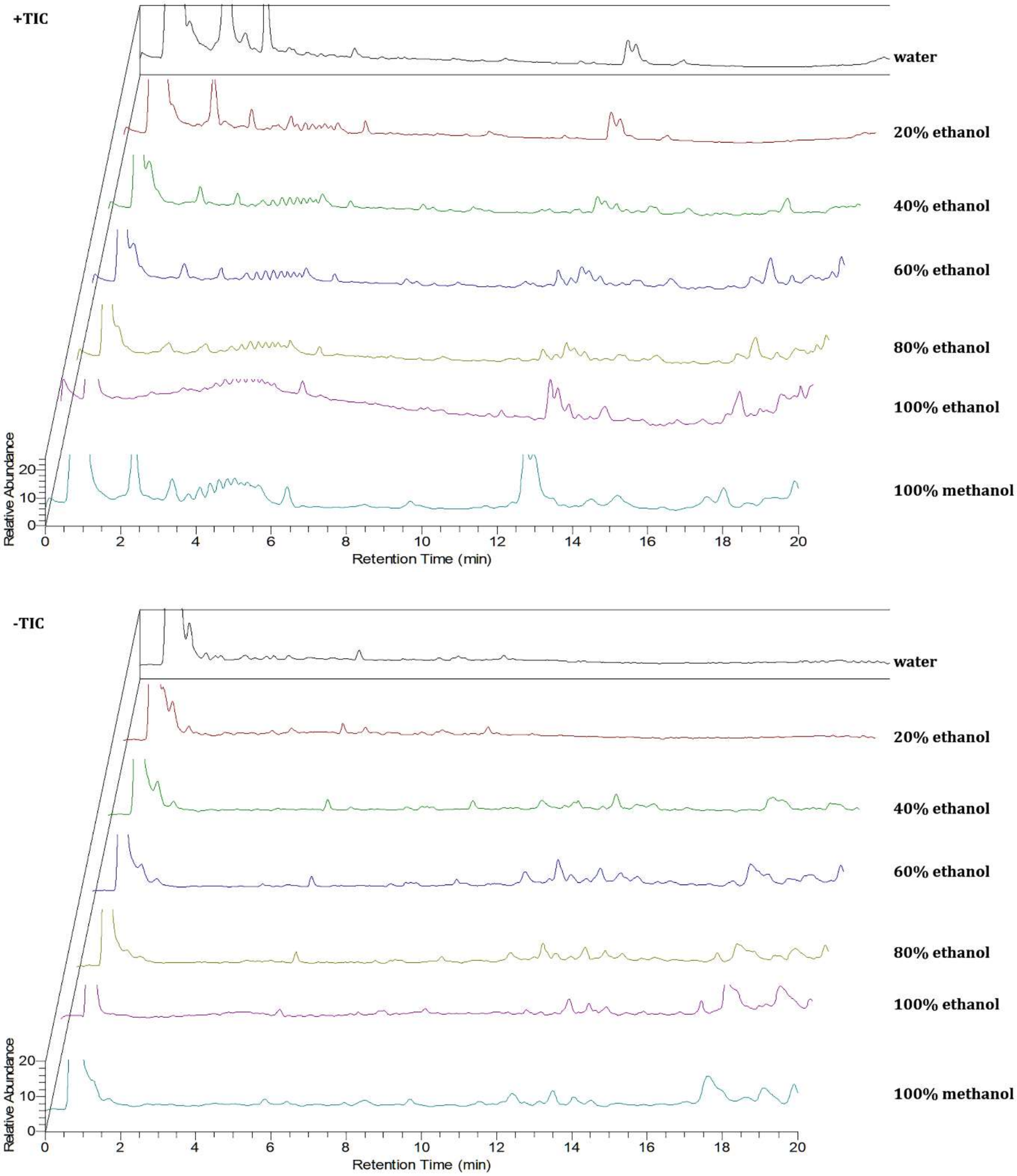

Figure S7. Comparison of total ion current chromatograms (TIC) of *S. crispa* extracted with different solvents in positive (above) and negative (below) ion modes (The mobile phase was composed of 0.1% formic acid aqueous solution (A) and acetonitrile (B), and the gradient elution program was 5–100% B at 0–20 min)

citricacidmonomethylester

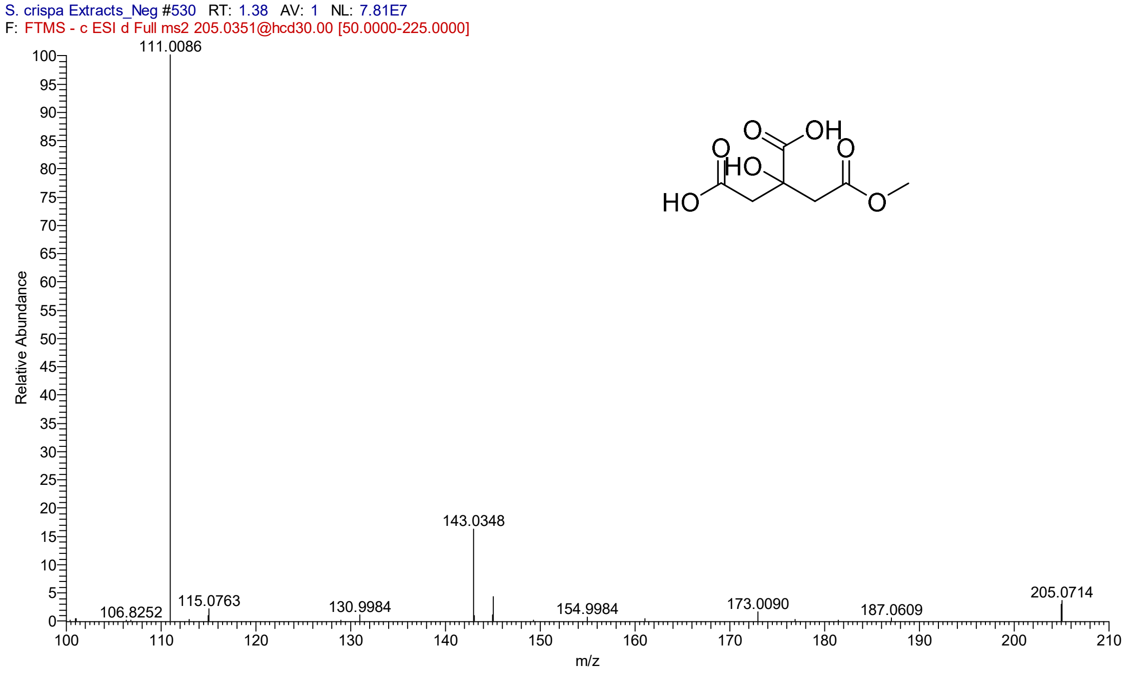

methylsuccinicacid

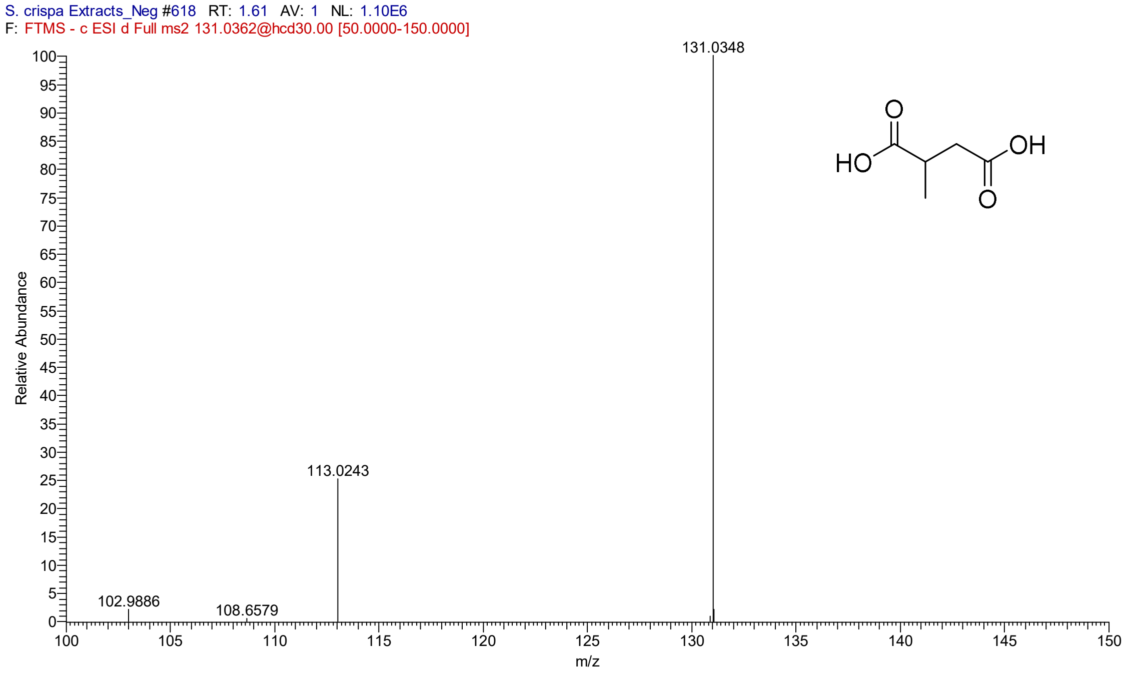

3-methylglutaricacid

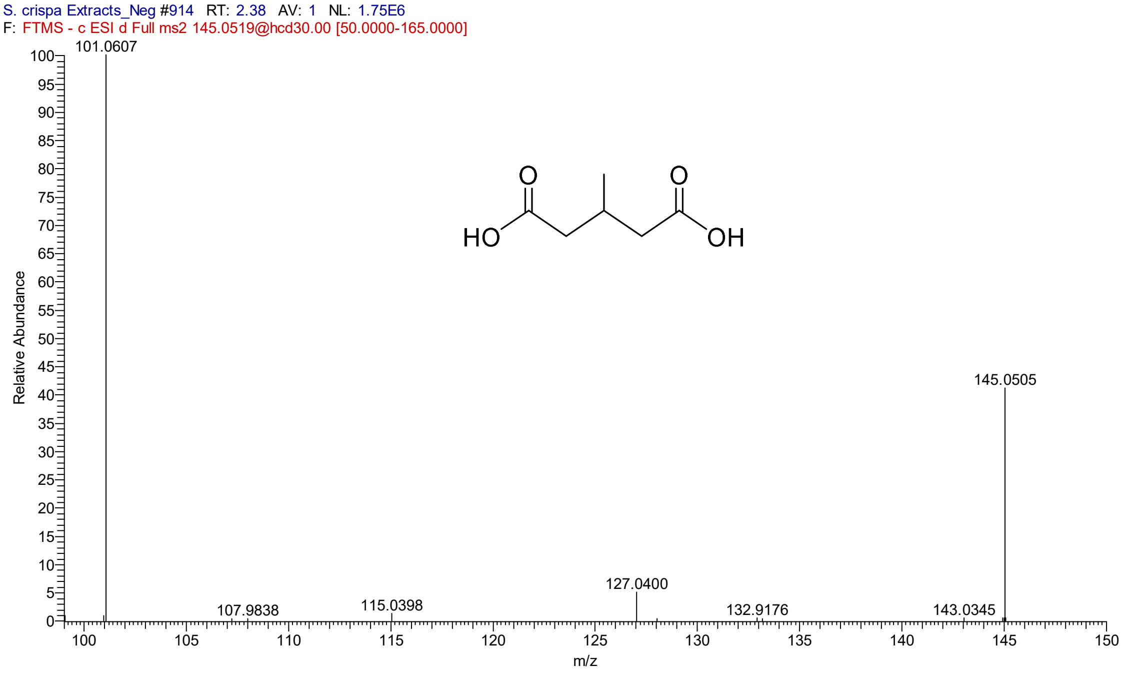

5′-deoxy-5′-methylthioadenosine

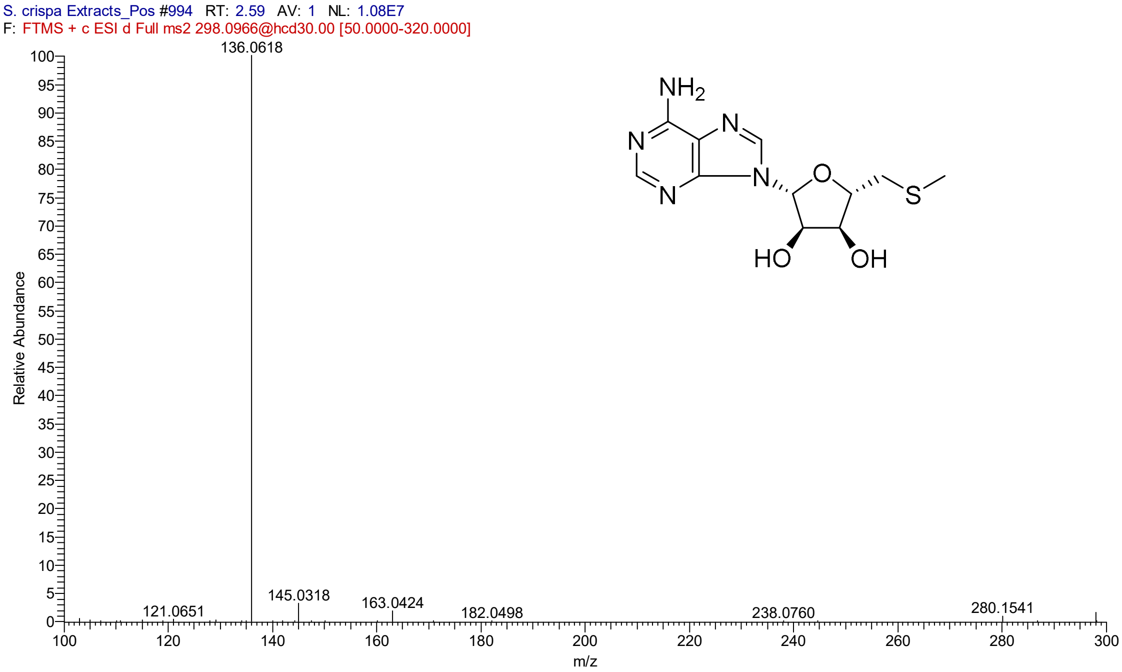

2-aminooctanedioicacid

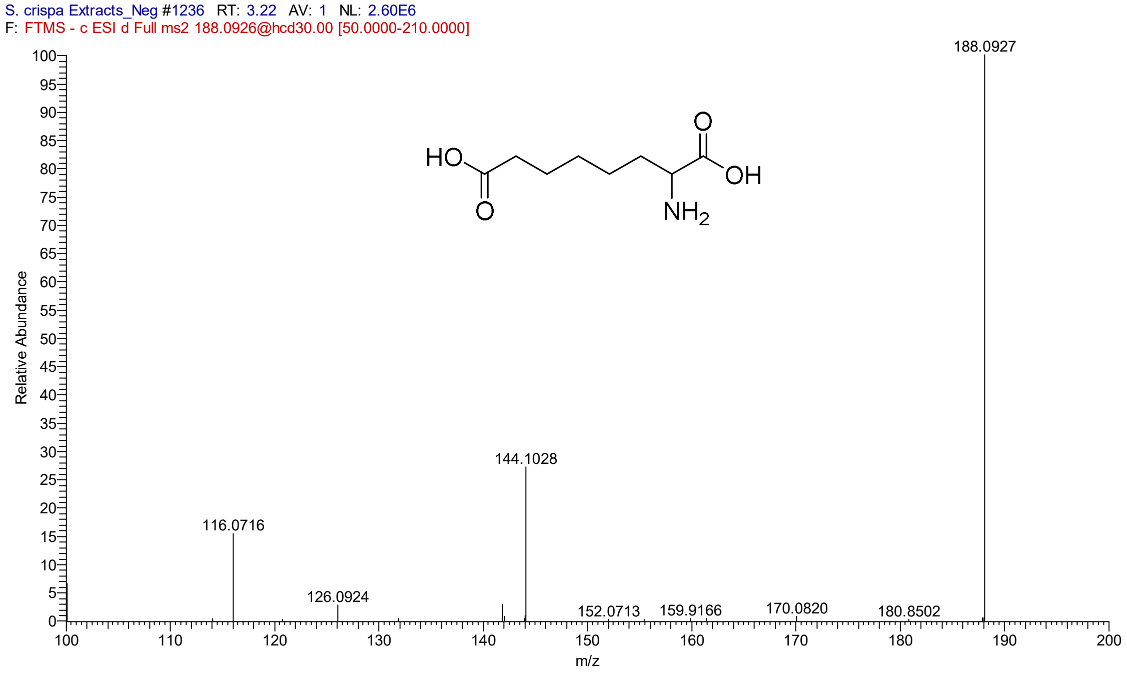

4-hydroxy-5,7-dimethoxy-1-isobenzofuranone

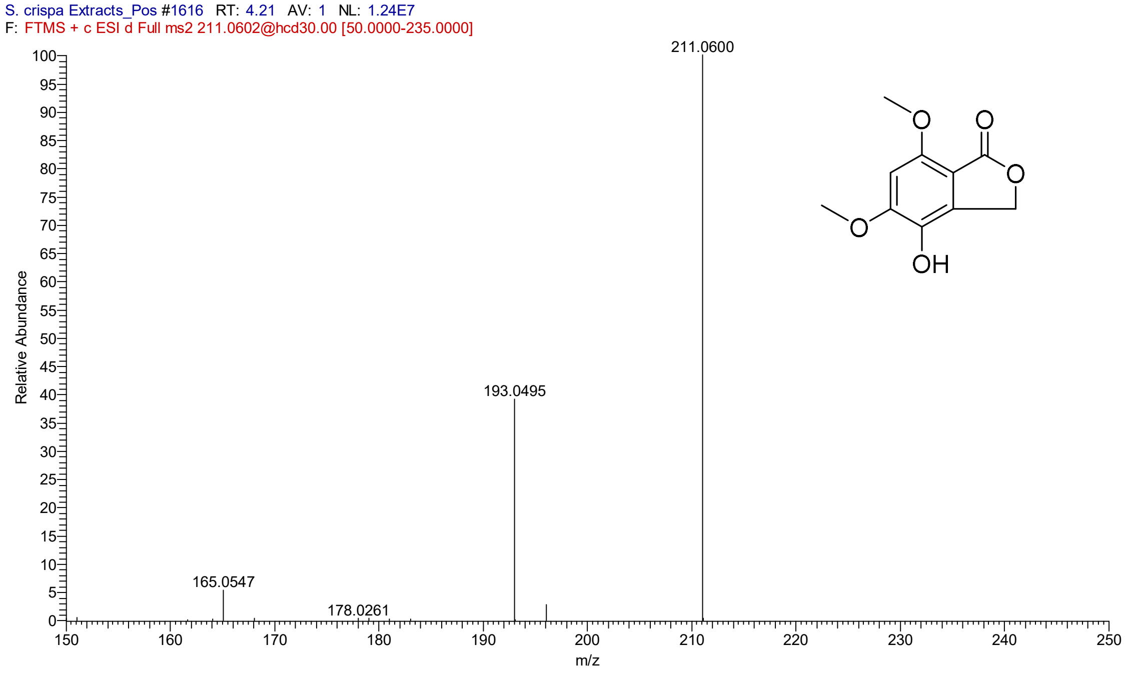

glucitol

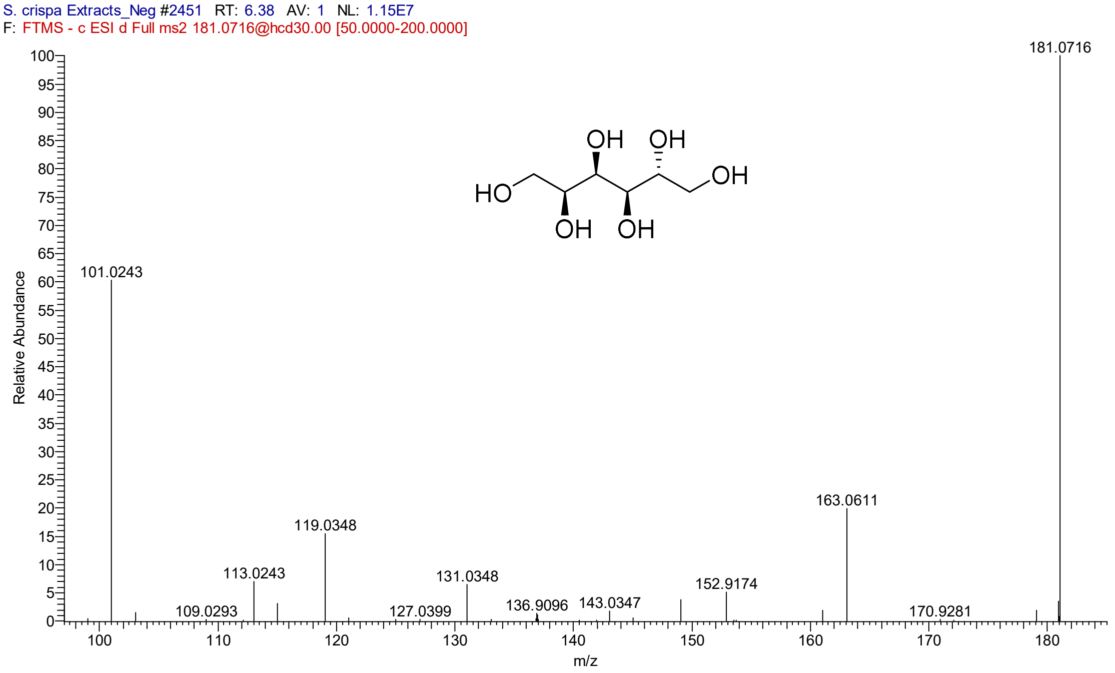

2-amino-1,3-dodecanediol

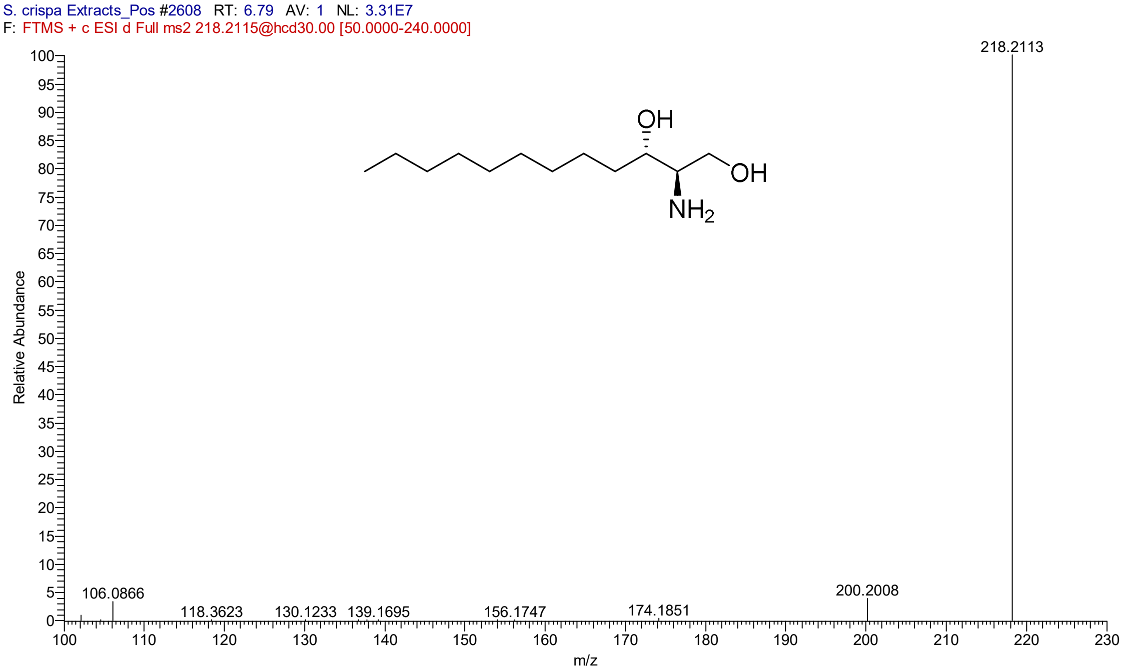

stellarinC

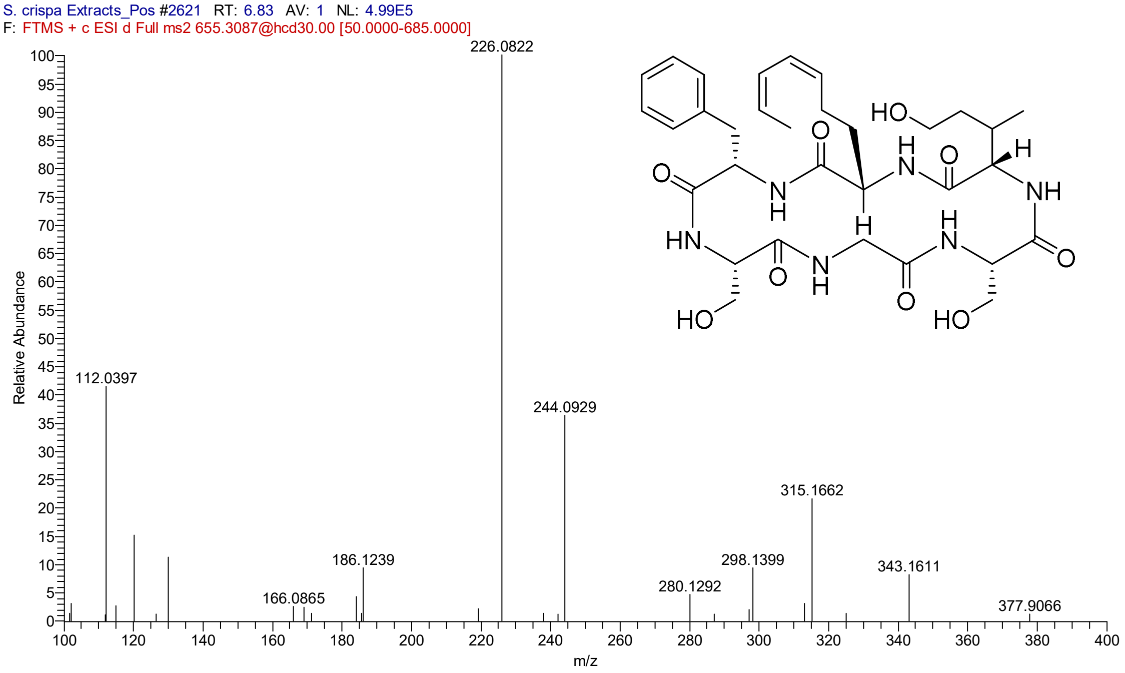

9-hydroxy-10,14-octadecadien-12-ynoicacid

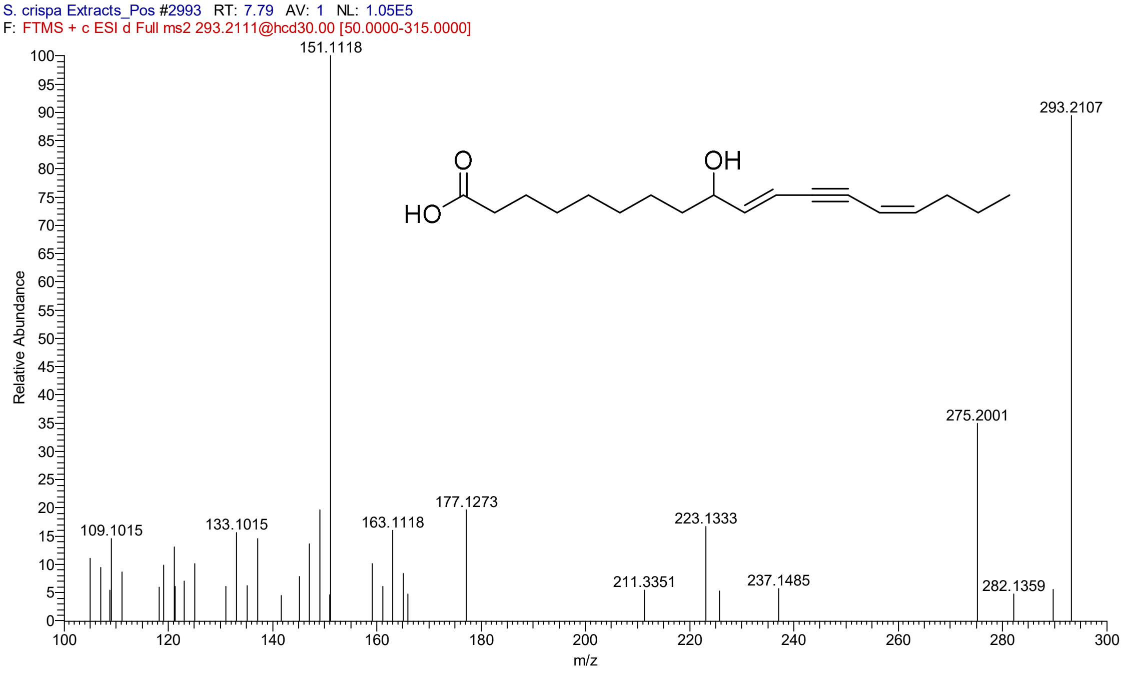

clitocybulolC

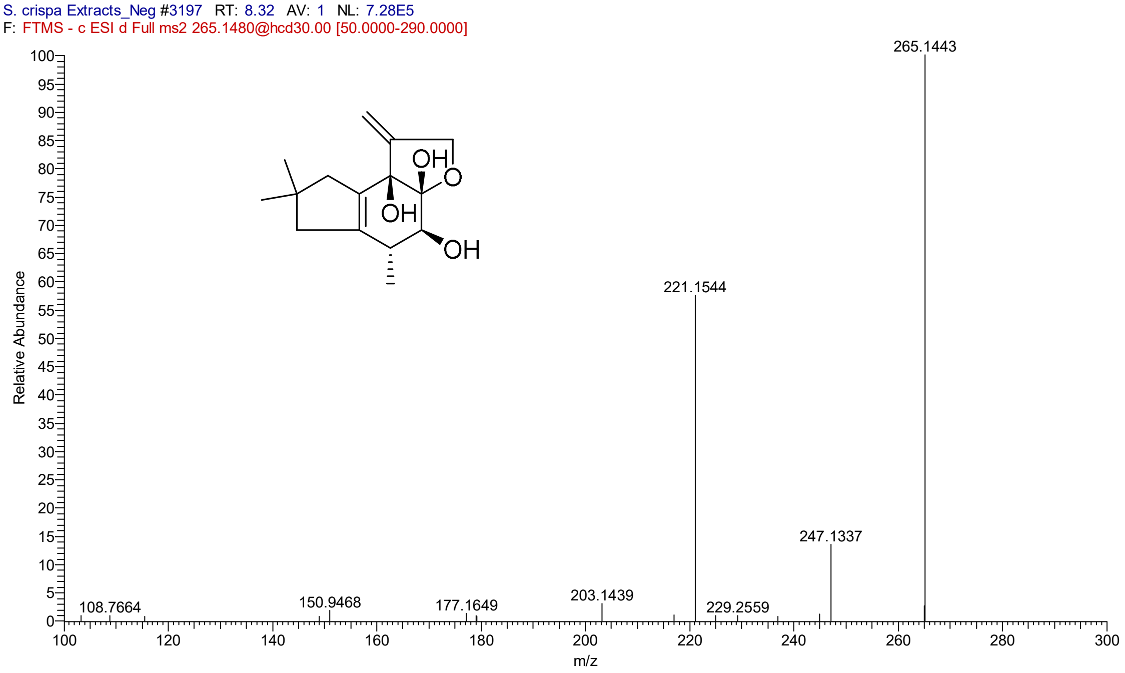

3β-hydroxy-11,12-O-isopropylidenedrimene

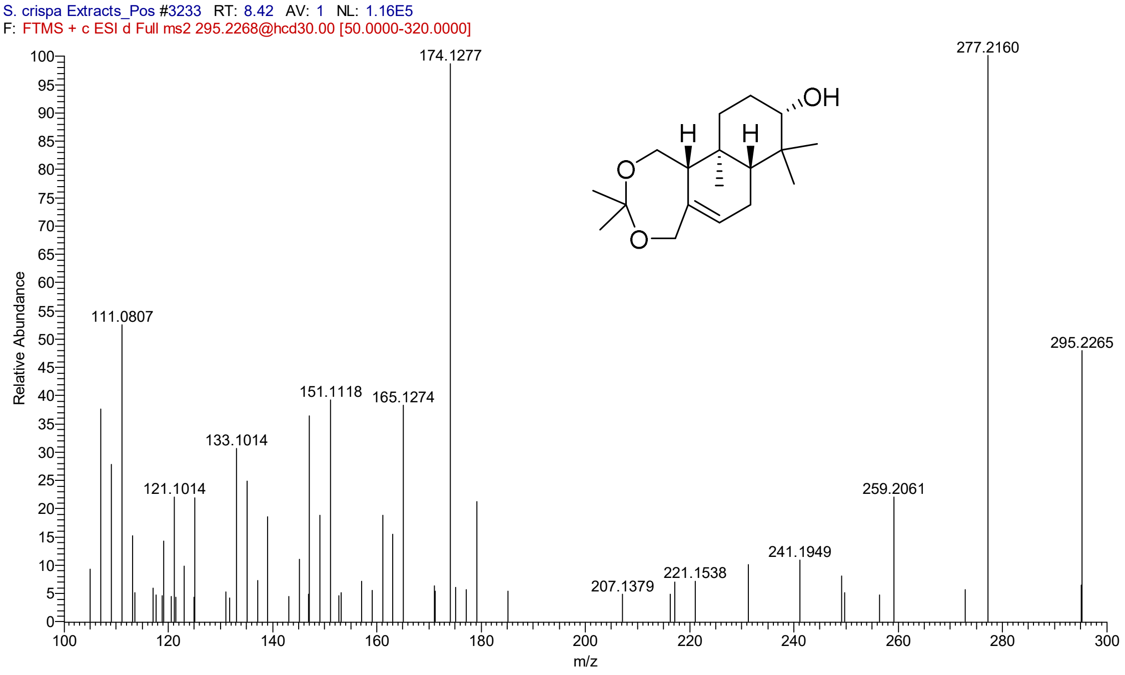

9,12,13-trihydroxy-15-octadecenoicacid

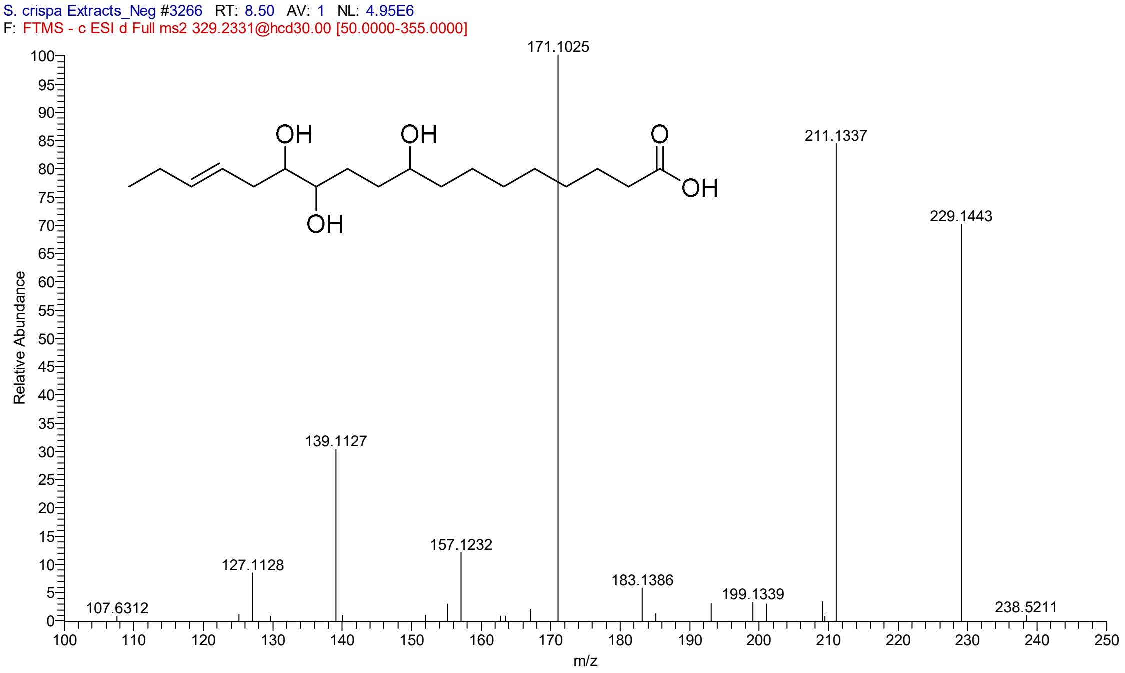

5,8-dihydroxy-9,12-octadecadienoicacid

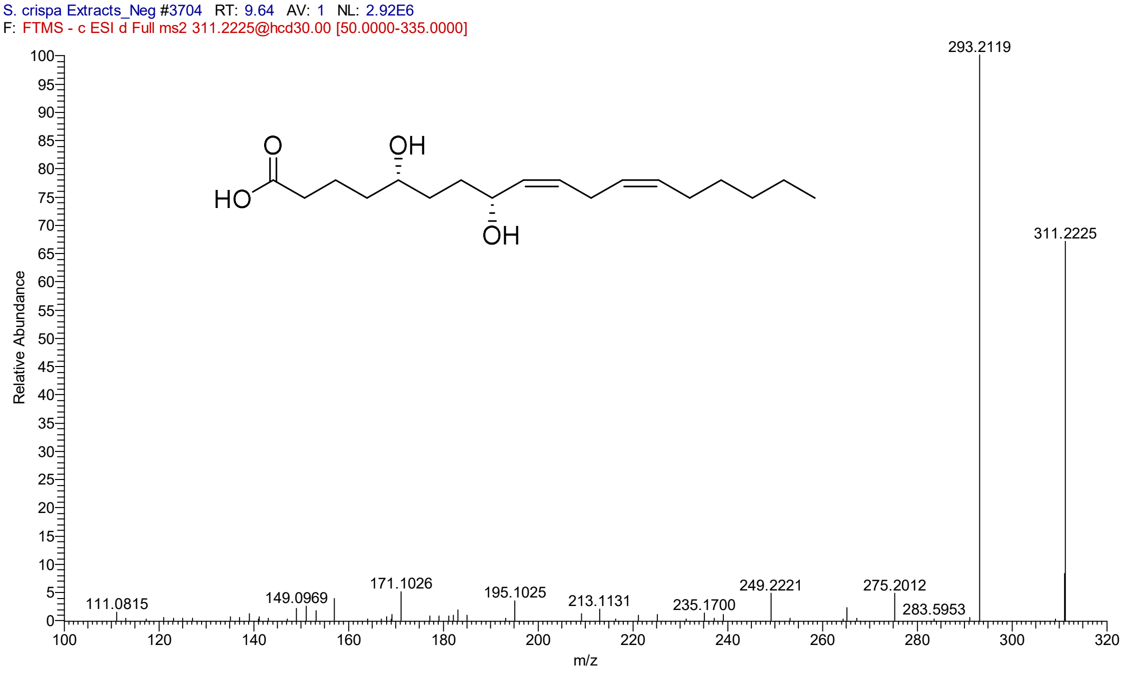

porrigenicacid

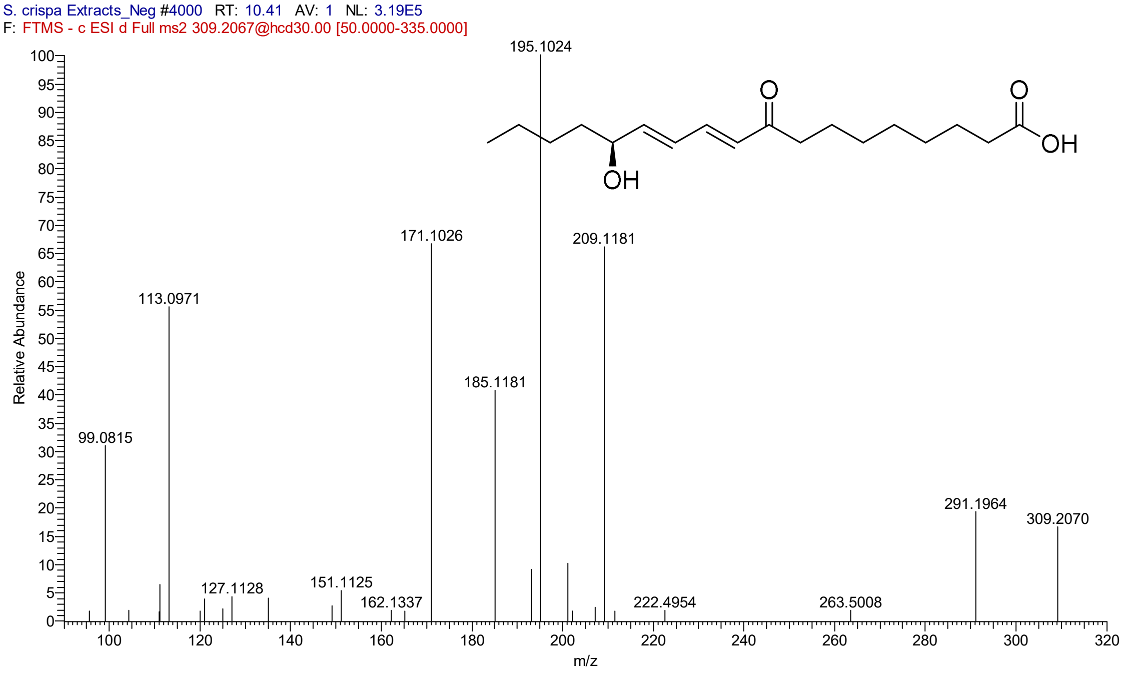

12-oxo-phytodienoicacid

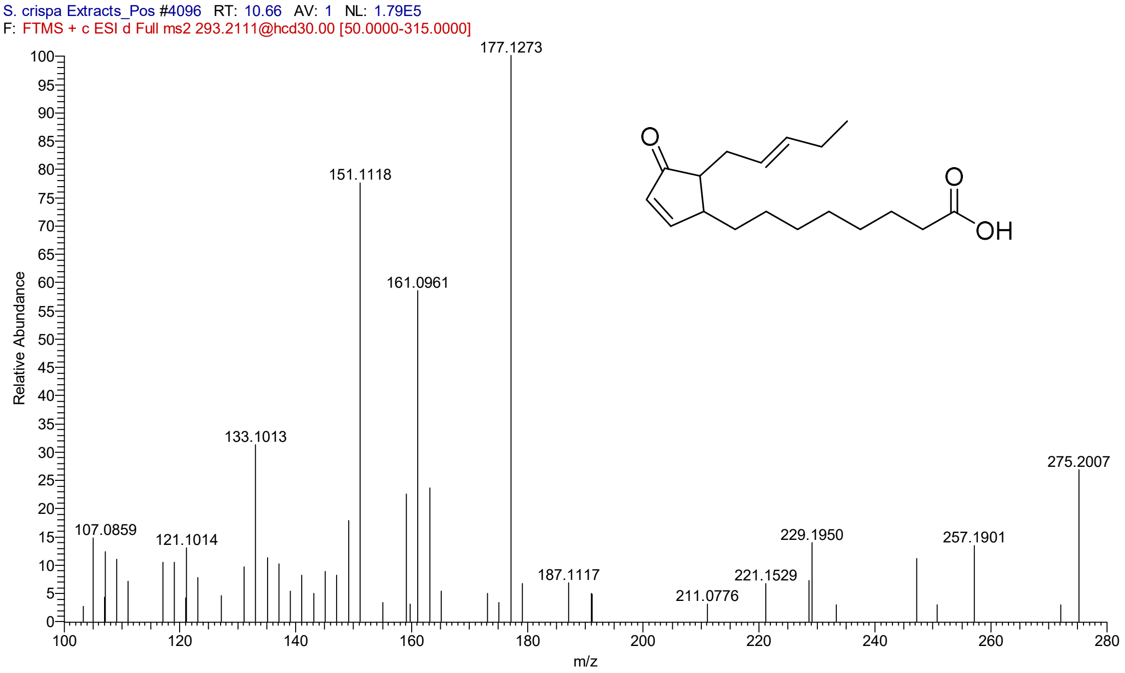

nicandroseE

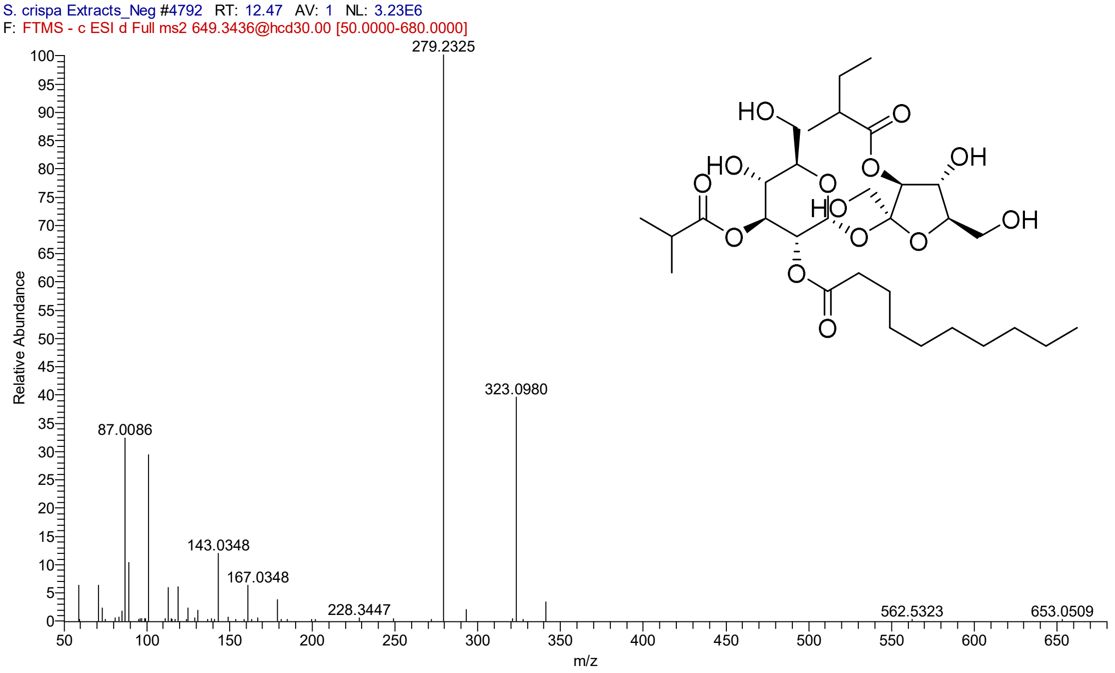

1,3-di(isobutoxycarbonyl)-2,4,4-trimethylpentane

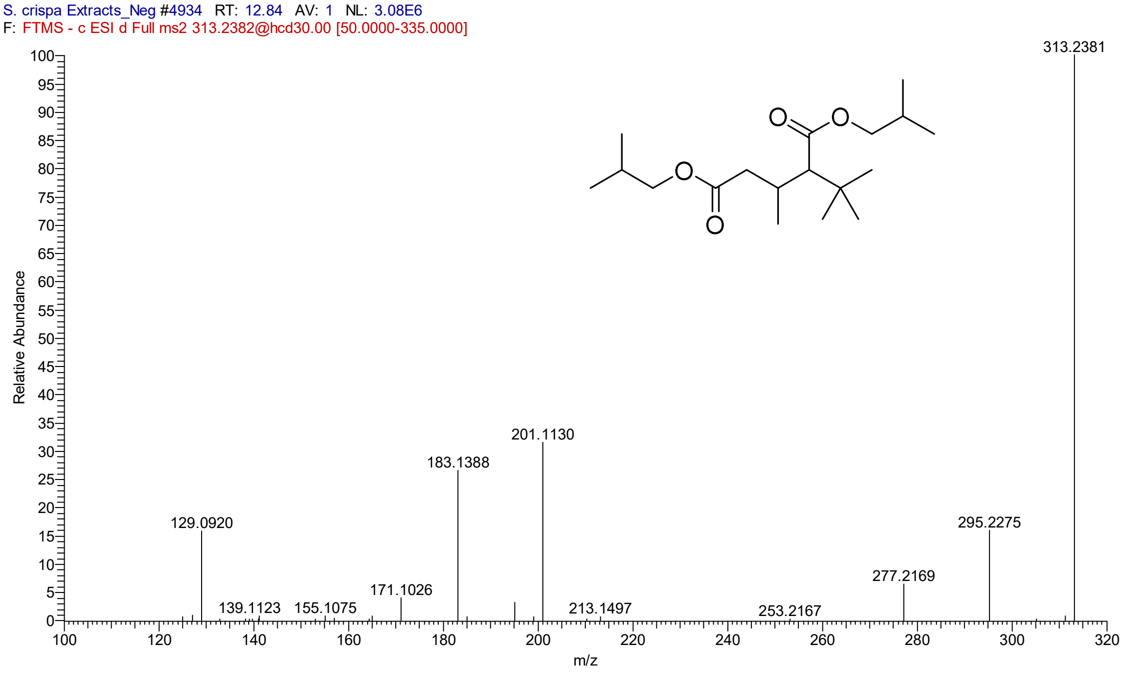

10-hydroxy-8,12-octadecadienoicacid

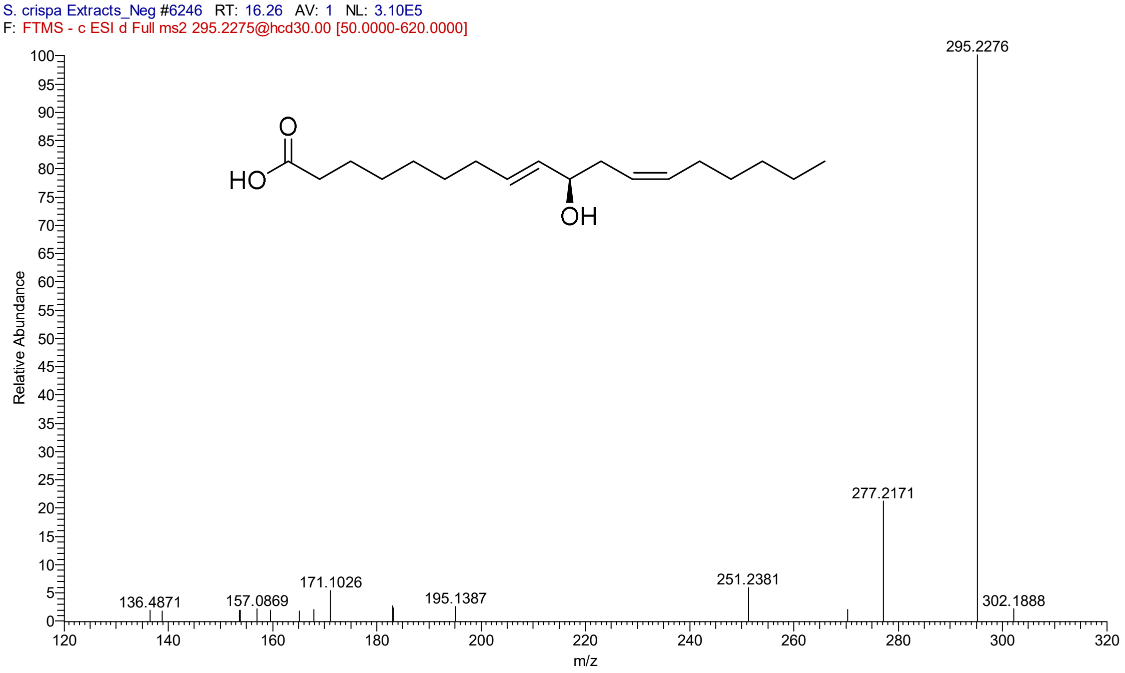

3-hydroxy-2-[[(9Z)-1-oxo-9-hexadecen-1-yl]oxy]propylβ-D-galactopyranoside

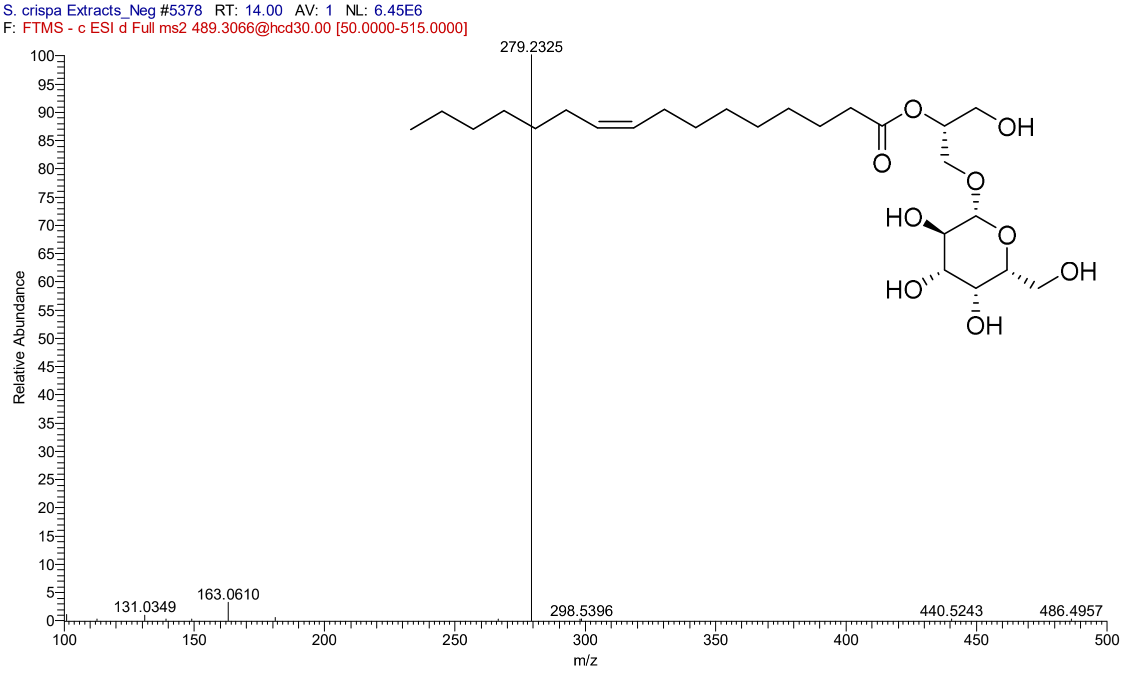

6,7-epoxystearicacid

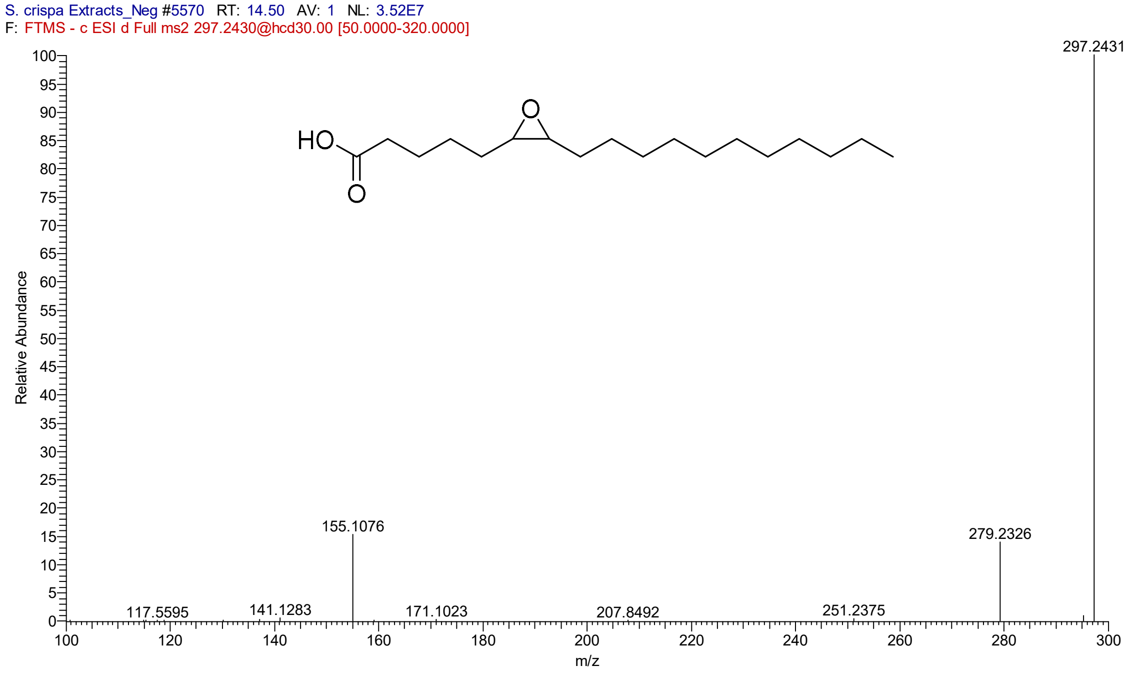

1-(9-octadecenoate)-β-D-glucopyranose

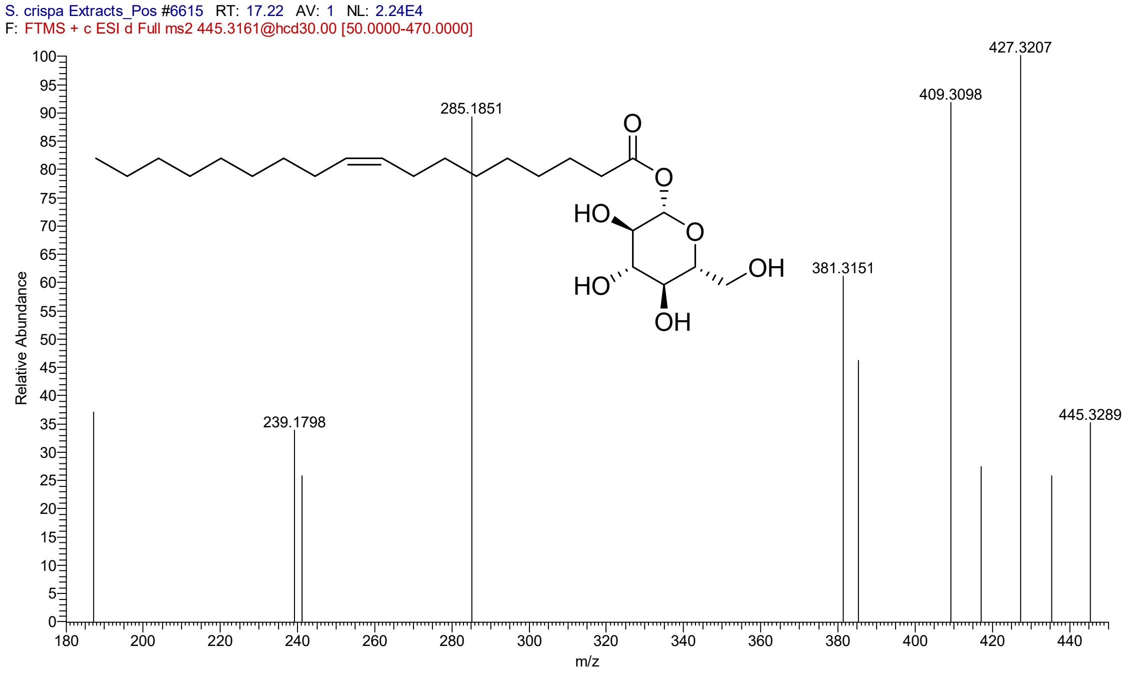

2,3-seco-2,3-dicarboxyplatanicacid

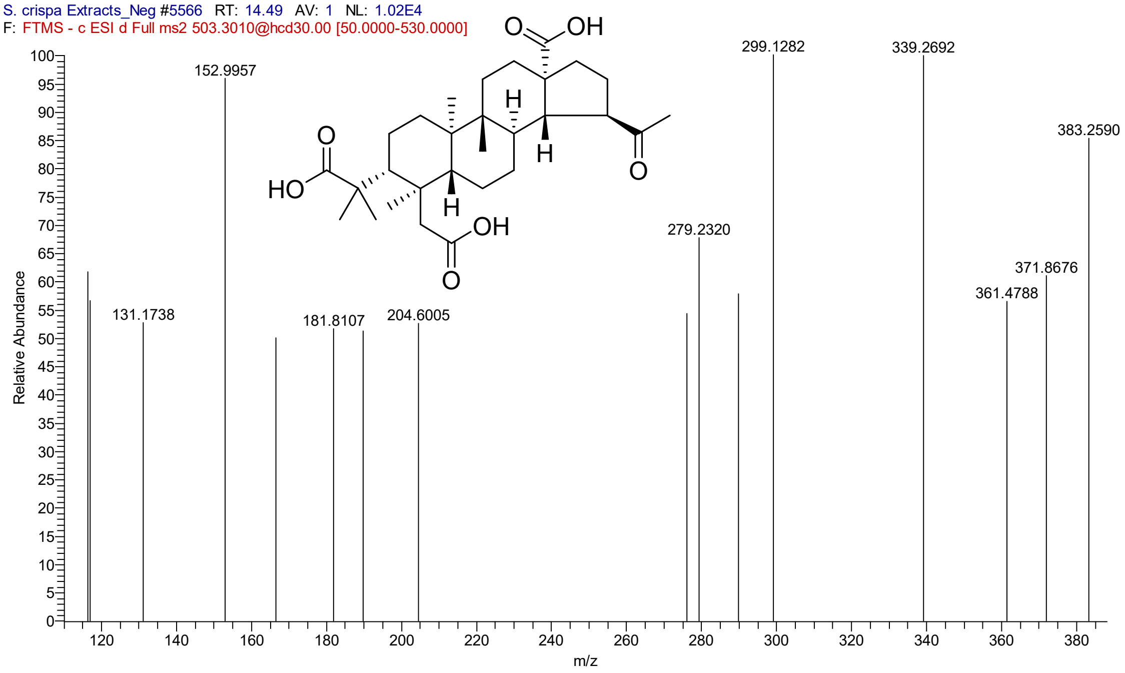

ganodermasideD

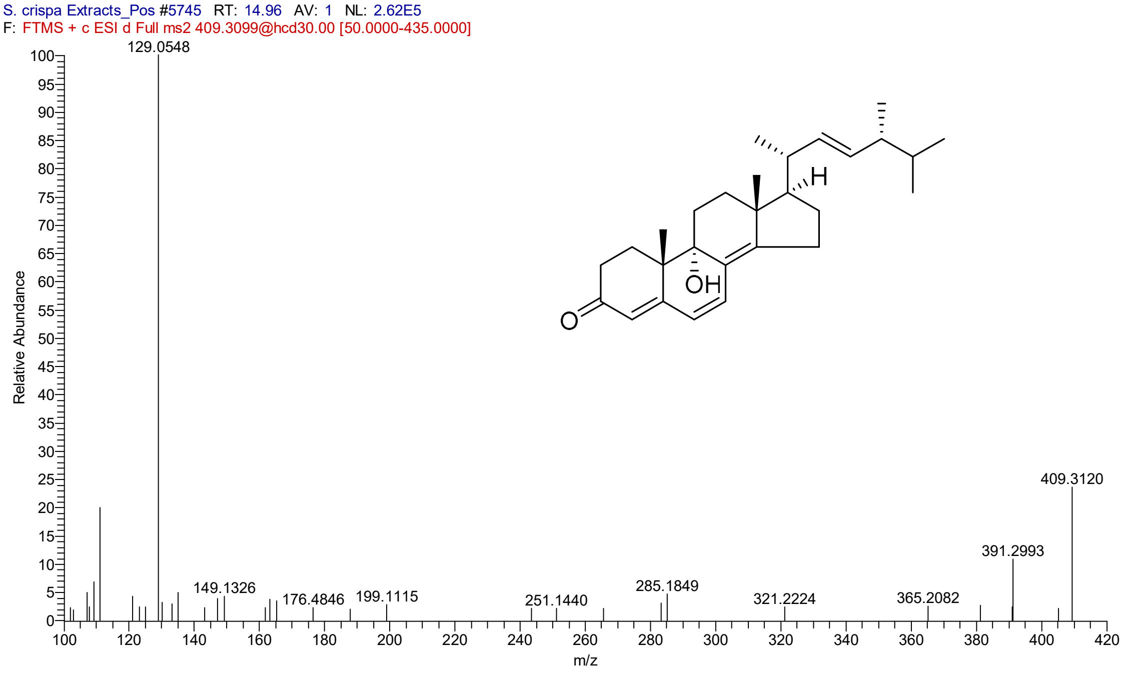

(5α,6α)-epoxy-ergosta-8(14),22-diene-3β,7β-diol

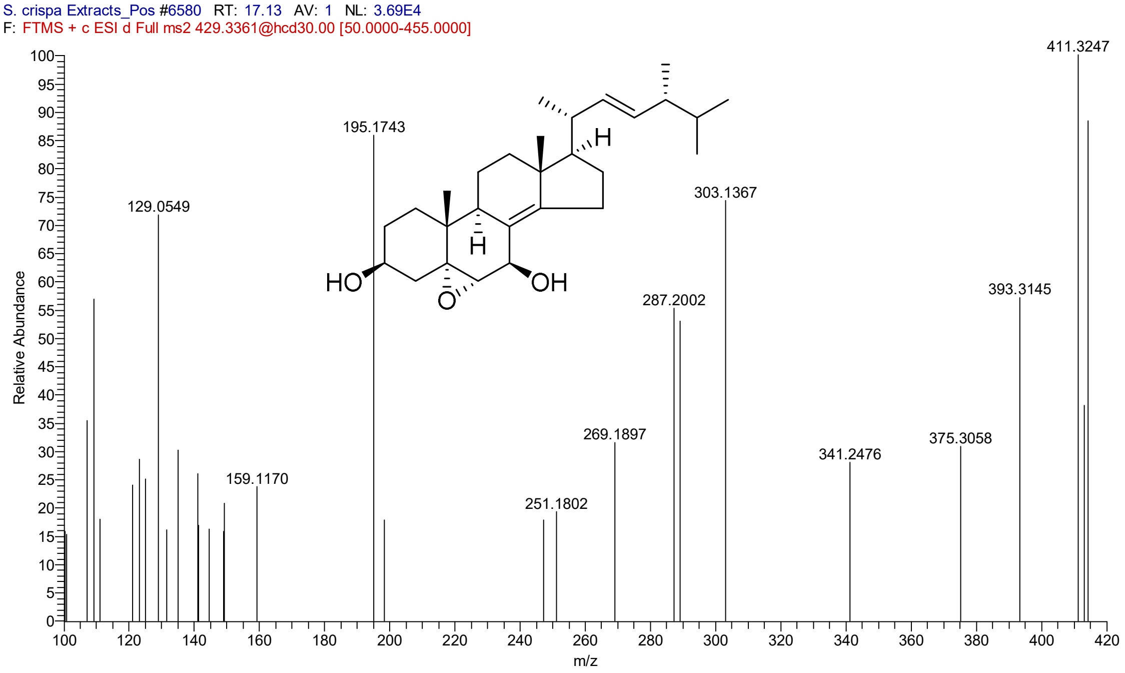

ergone

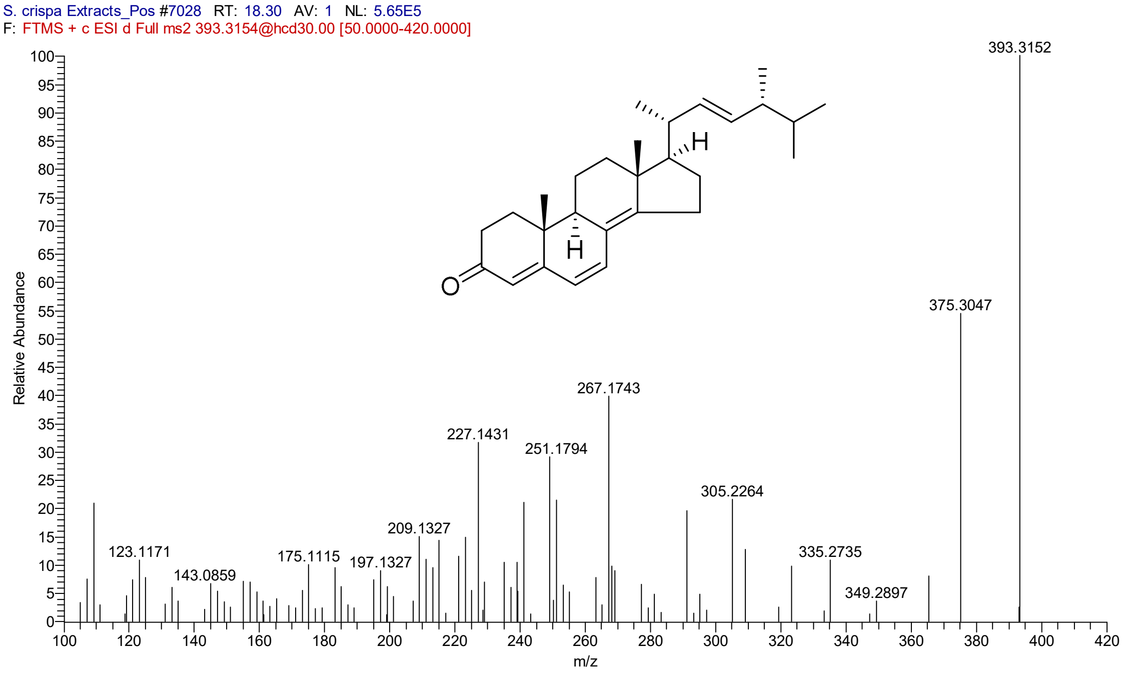

linoleicacid

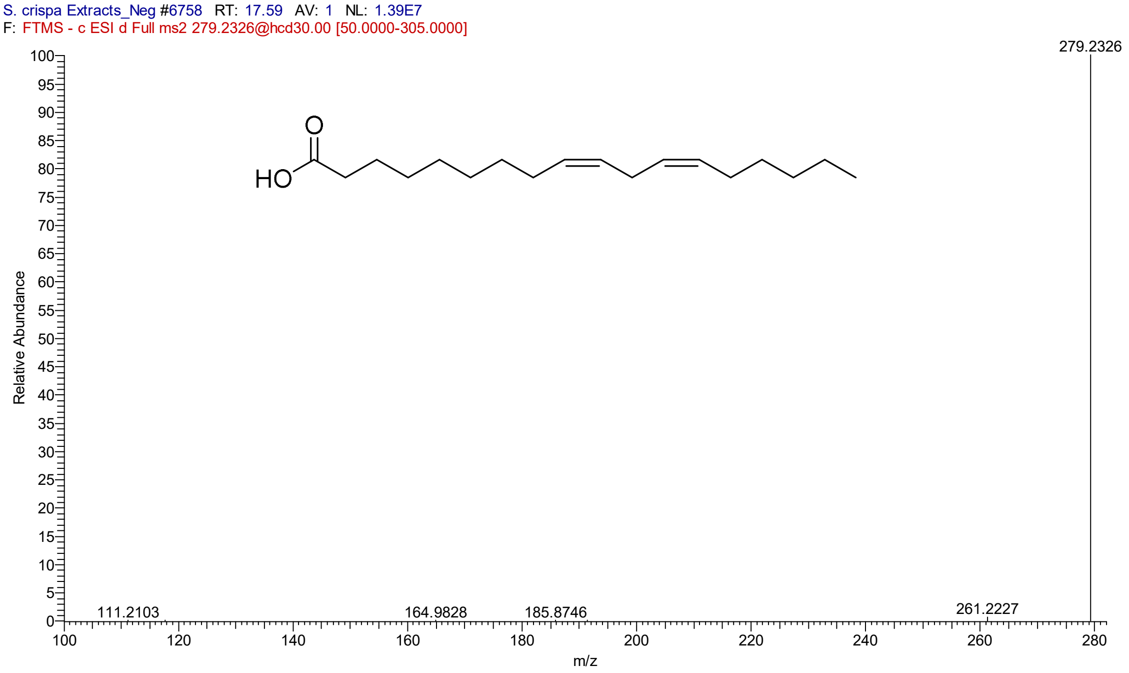

oleamide

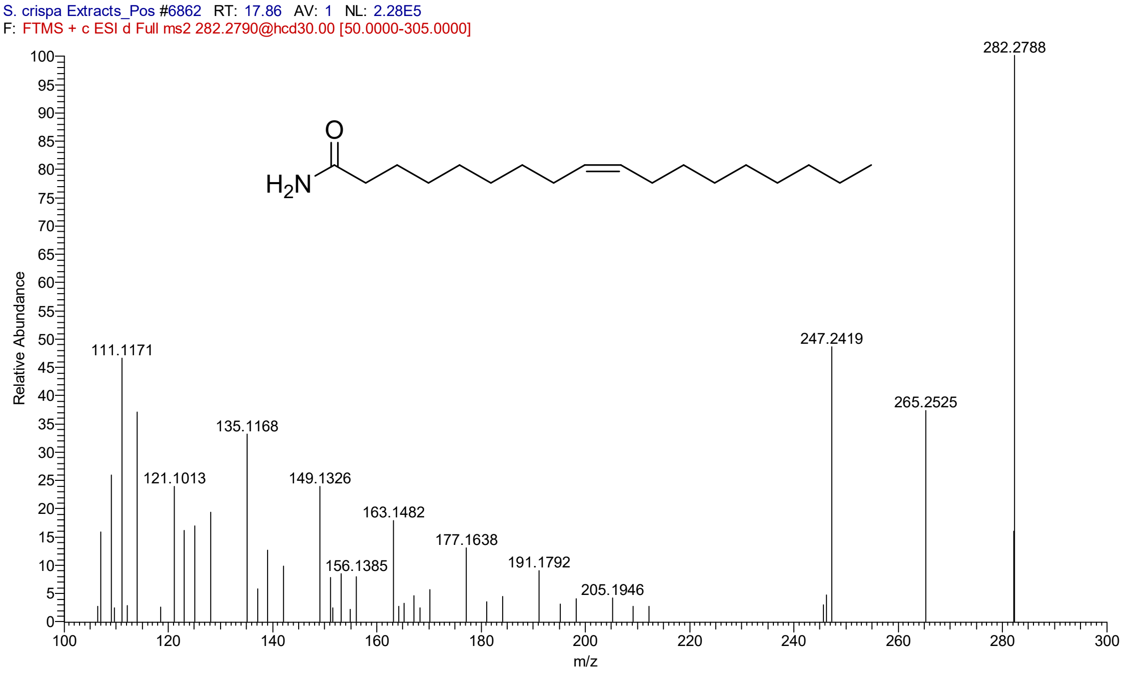

ergosterolperoxide

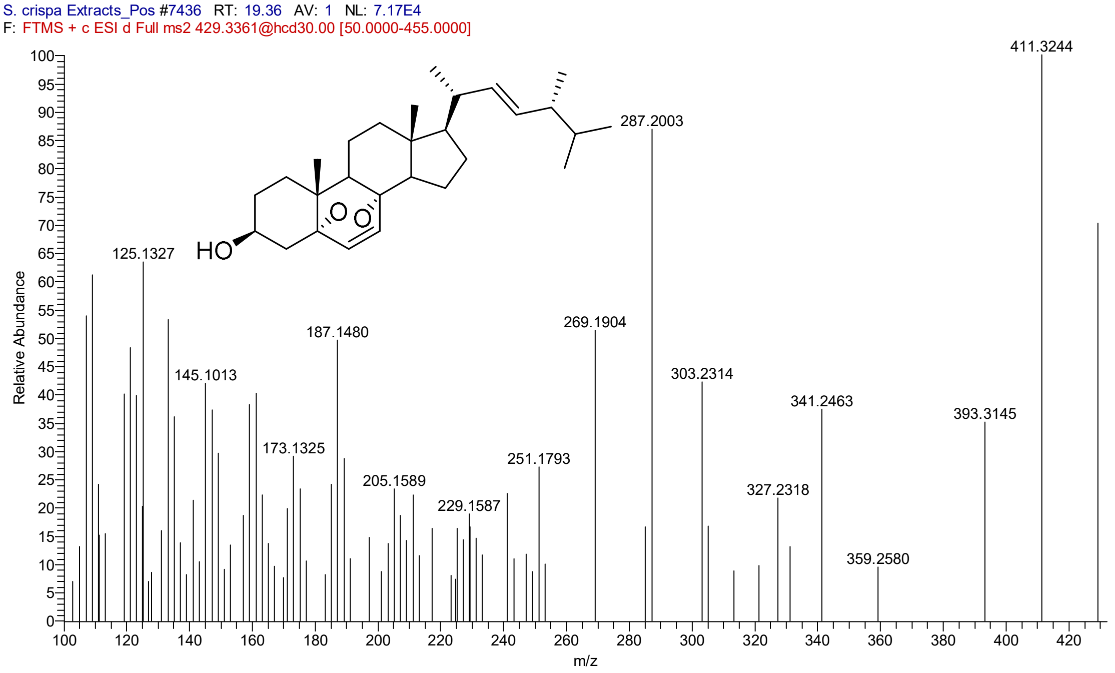

ergosta-5,8,22-triene-3,11-dione

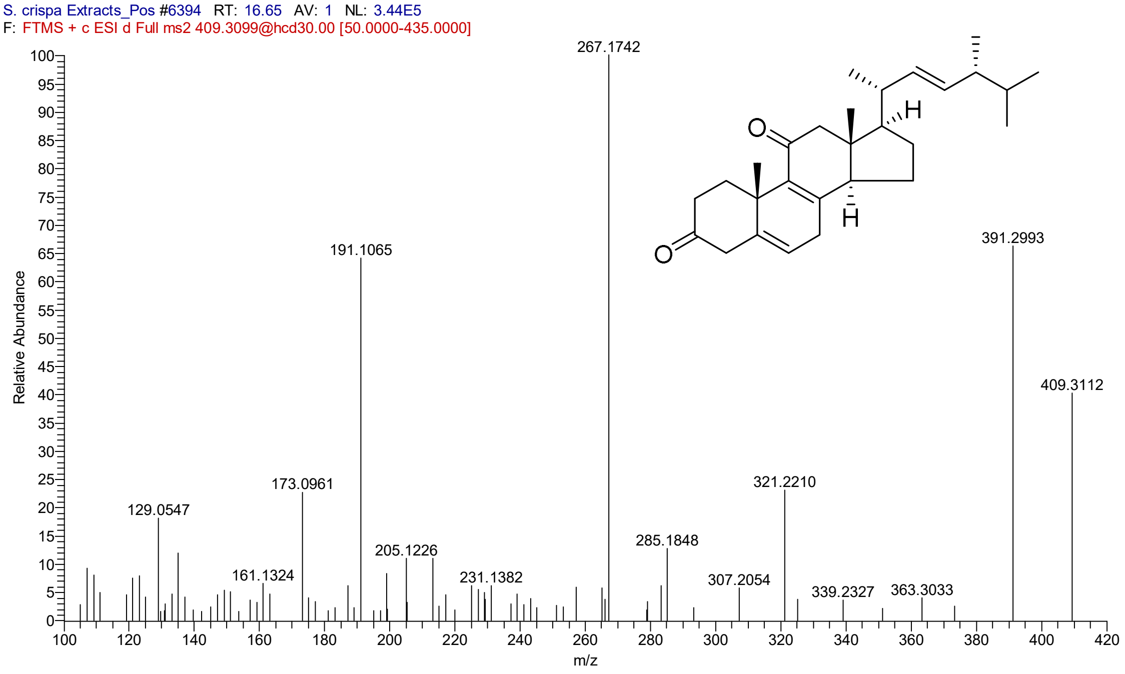

thraustochytrosideA

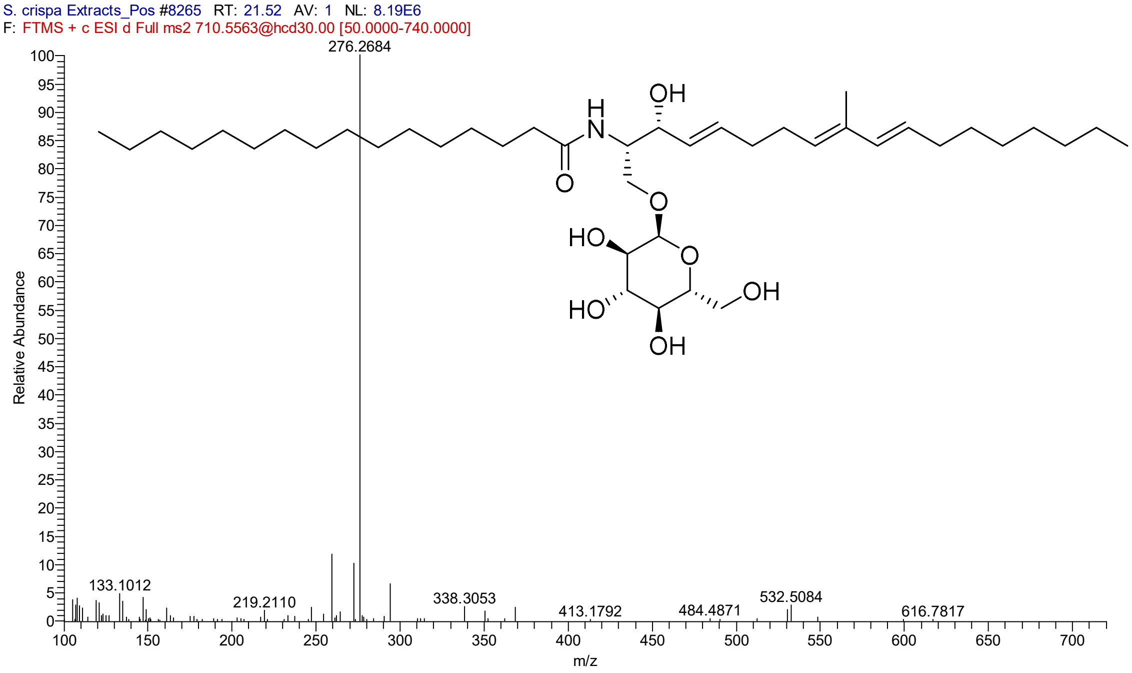

erucamide

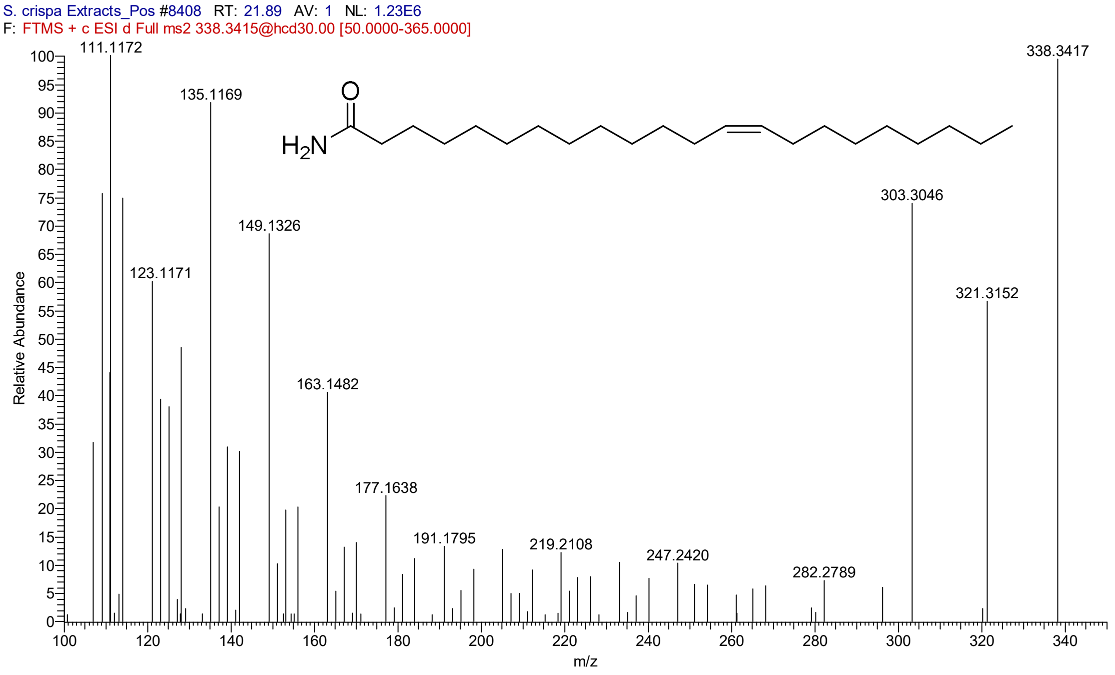

1-(hydroxymethyl)-2[(1-oxohexadecyl)oxy]ethyl-ester-9,12-heptadecadienoicacid

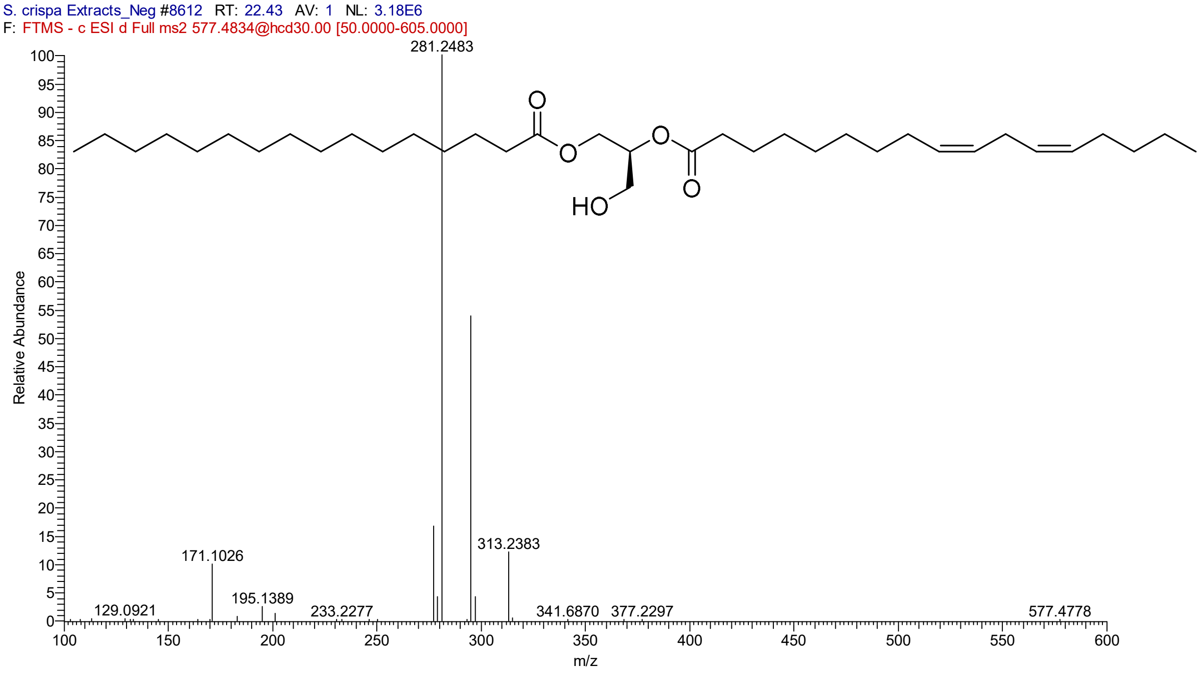

Figure S8. Chemical structures and available raw MS² spectra of some components identified from *S. crispa*
